# Vitexin confers HSF-1 mediated autophagic cell death by activating JNK and ApoL1 in colorectal carcinoma cells

**DOI:** 10.18632/oncotarget.20113

**Published:** 2017-08-10

**Authors:** Monika Bhardwaj, Souren Paul, Rekha Jakhar, Imran Khan, Ji In Kang, Ho Min Kim, Jong Won Yun, Seon-Jin Lee, Hee Jun Cho, Hee Gu Lee, Sun Chul Kang

**Affiliations:** ^1^ Department of Biotechnology, Daegu University, Kyoungsan, Kyoungbook, Republic of Korea; ^2^ Disease Molecule Biochemistry Laboratory, Graduate School of Medical Science and Engineering (GSMSE), KAIST, Yuseong-gu, Daejeon, Republic of Korea; ^3^ Immunotherapy Convergence Research Center, Korea Research Institute of Bioscience and Biotechnology, Daejeon, Republic of Korea; ^4^ Department of Biomolecular Science, University of Science and Technology (UST), Daejeon, Republic of Korea

**Keywords:** vitexin, HSF-1, ApoL1, autophagic cell death, colorectal carcinoma

## Abstract

Heat shock transcription factor-1 (HSF-1) guards the cancerous cells proteome against the alterations in protein homeostasis generated by their hostile tumor microenvironment. Contrasting with the classical induction of heat shock proteins, the pro-oncogenic activities of HSF-1 remains to be explored. Therefore, cancer's fragile proteostatic pathway governed by HSF-1 could be a potential therapeutic target and novel biomarker by natural compounds. Vitexin, a natural flavonoid has been documented as a potent anti-tumor agent on various cell lines. However, in the present study, when human colorectal carcinoma HCT-116 cells were exposed to vitexin, the induction of HSF-1 downstream target proteins, such as heat shock proteins were suppressed. We identified HSF-1 as a potential molecular target of vitexin that interact with DNA-binding domain of HSF-1, which inhibited HSF-1 oligomerization and activation (*in silico*). Consequently, HSF-1 hyperphosphorylation mediated by JNK operation causes transcriptional inactivation of HSF-1, and supported ROS-mediated autophagy induction. Interestingly, in HSF-1 immunoprecipitated and silenced HCT-116 cells, co-expression of apolipoprotein 1 (ApoL1) and JNK was observed which promoted the caspase independent autophagic cell death accompanied by p62 downregulation and increased LC3-I to LC3-II conversion. Finally, *in vivo* findings confirmed that vitexin suppressed tumor growth through activation of autophagic cascade in HCT-116 xenograft model. Taken together, our study insights a probable novel association between HSF-1 and ApoL-1 was established in this study, which supports HSF-1 as a potential target of vitexin to improve treatment outcome in colorectal cancer.

## INTRODUCTION

Cells encounter stress from various environmental cues which causes profound alterations in proteome homeostasis known as proteotoxic stress. They are well equipped with sophisticated adaptive mechanisms to combat these stresses. At molecular level, heat shock response (HSR), a ubiquitous transcriptional response driven by heat shock transcription factor (HSF-1), governs the expression of heat shock proteins (Hsps) for cytoprotection [1]. HSF-1 exists as an inactive monomer, bound by heterocomplex structure of Hsp90 and Hsp70 [2, 3]. During acute proteotoxic stress, HSF-1 accumulates in the nucleus, oligomerizes and become transcriptionally active to increase Hsps and other cytoprotective target gene levels to survive proteotoxic stress. HSF-1 is transiently activated and attenuates in parallel with alleviation of stress [4]. In contrast, HSF-1 is constitutively active in cancer cells and plays a multifaceted role in carcinogenesis, including expression of atypical levels of Hsps, malignant transformation, and others, hence identified as a biomarker for cancer prognosis [5, 6].

Macroautophagy (hereafter referred autophagy) is a well conserved, highly orchestrated lysosomal catabolic process in eukaryotes that facilitates the degradation or recycling of superfluous or impaired cellular constituents via autophagolysosome formation [7]. The dual role assayed by autophagy in cancer cells, which bifurcates into cellular death or survival pathway, function in a context-dependent manner [8]. During starvation, autophagy promotes cell survival by providing biofuel from degraded macromolecules, whereas its role remains paradoxical in cancer cells [9]. Autophagy can be activated by variety of extracellular stimulus including chemicals, pharmaceuticals, pro-oxidant compounds etc. [10], which overexpress tumor suppressor genes and promotes caspase-independent cell death [11].

HSR and autophagy represent two ends of the protein homeostasis spectrum, which are likely to complement each other during cellular stress. Earlier studies reported that Hsp70 overexpression prevented p62/sequestosome/SQSTM1 degradation and decrease LC3-II protein expression, indicating autophagy regulation by HSR. [12]. HSF-1 negatively regulates autophagy via activation of Akt-mTOR pathway [13]. Additionally, Hsp70 overexpression inhibits OSU-03012 induced autophagy and switches HCT-116 and U251 cells from degradation to protein synthesis phase [14].

Bcl-2 homology domain (BH3)-only family proteins are important regulators of type I programmed cell death (PCD) apoptosis; however their role for type II PCD autophagy remains elusive. BH3 domain exhibits functional significance in PCD as, it is the only domain retained by BH3-only proteins and found conserved across all Bcl-2 family members [15]. Apart from their role in apoptosis, Bcl-2 family proteins play a crucial role in regulating autophagy mediated cell death (ACD). In the context of molecular regulators that dictate ACD, a novel *bona fide* BH3-only pro-death protein apolipoprotein L1 (ApoL1) has been characterized for induction of ACD independent of caspase-mediated apoptosis which can be a general autophagy mediator [16].

**Figure 1:**
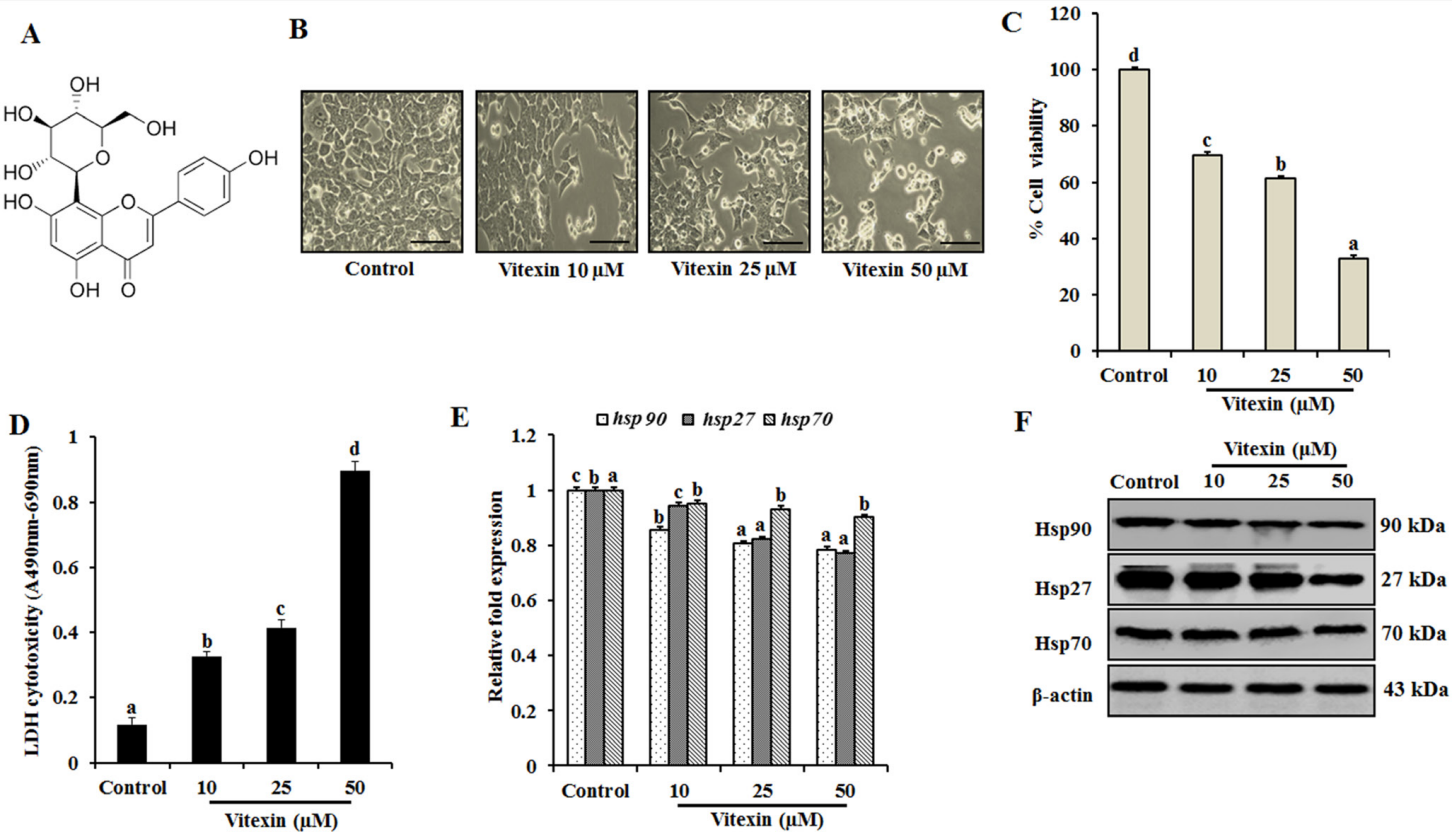
Vitexin inhibits cell proliferation and exerts cytotoxic effects against human colorectal cancer cells (**A**) Chemical structure of vitexin. (**B**) HCT-116 cells were treated with 10, 25 and 50 μM concentration of vitexin for 24 h. The morphological changes in HCT-116 cells were studied using light microscopy. Scale bar 0.1 mm. Cell viability and toxicity of various concentrations of vitexin was assayed by (**C**) MTT and (**D**) LDH assays in HCT-116 cells, as compared to the control cells. (**E**) Expression profiles of *hsp90*, *hsp27*, and *hsp70* mRNA were assayed by real-time PCR. Data represents fold change versus control. (**F**) Representative immunoblot analysis of Hsp90, Hsp27 and Hsp70 proteins were determined by whole cell lysates. The data represents mean ± SD of three independent experiments, *n* = 3. Values with different letters (a-d) differ significantly from each other (*p* < 0.05).

Flavonoids, the active polyphenolic constituents, found ubiquitously in various dietary foods display therapeutic effects against cancer by inducing programmed cell death with minimized side effects [17]. Vitexin (apigenin-8-C-D glucopyranoside) (Figure 1A), an apigenin flavone glucoside, has been reported to exhibit diverse biological activities [18–21], and exerts anti-cancer efficacy against various cancer lines through apoptotic and autophagic cell death, by targeting several transcription factors, which are requisite for cancer progression [22].

Given evidences for the significant impairment of human malignant cell lines proliferation by knockdown of HSF-1 in contrast with minimal effect on normal cell line, HSF-1 could be a promising target for the cancer therapy. HCT-116 cells possess about 10 fold increases of HSF-1 levels than normal cells, which is responsible for mediating colorectal cancer progression [23]. In the present report, we selected HCT-116 cell line and attempted to elucidate the role of vitexin on colorectal carcinoma (CRC). Vitexin was identified for transcriptional inactivation of HSF-1 and abolishing synthesis of Hsps. Vitexin coupled cyanogen bromide sepharose beads demonstrated an association between vitexin and HSF-1. Evidences that vitexin hinders CRC growth by c-Jun N-terminal kinase (JNK) and ApoL1-mediated ACD are also presented. Finally, in nude mice HCT-116 cells xenograft model, vitexin resulted in repression of tumor growth suggesting HSF-1 as potential therapeutic target.

## RESULTS

### Vitexin blocks induction of stress-responsive proteins

To investigate cytotoxic effect of vitexin, HCT-116 cells were treated with vitexin (10, 25 and 50 μM) for 24 h. Morphological analysis reveal that control cells were well adhered, showing normal morphology of HCT-116 cells. Tumor cells treated with vitexin caused remarkable changes with majority of cells to become shrink and show distorted morphology characteristics of programmed cell death (Figure 1B), with significant cell death as evidenced by reduction in cell viability and enhanced LDH activity (Figure 1C, 1D). The colony formation and soft agar assay showed inhibited colony formation with vitexin treatment (Supplementary Figure 1), whereas it did not exert cytotoxicity on CCD-112 CoN fibroblast cell line (Supplementary Figure 2).

Cancer cells experience autonomous sources of stress which dramatically increase the production of Hsps when compared to normal cells. Therefore, natural products can disrupt the protein homeostasis by inhibiting Hsps production and prohibiting their capacity to compensate for tumor cell survival [24]. Induction of hsp90 and hsp27 mRNA levels was reduced with no significant differences in hsp70 mRNA levels were observed in various concentrations of vitexin (Figure 1E). Consistent with previous result, dose-dependent reduction of Hsp90 and Hsp27 proteins with no prominent changes in Hsp70 expression was observed after vitexin treatment (Figure 1F; Supplementary Figure 3A).

### Vitexin induces nuclear localization of HSF-1 and hampers HSF-1 oligomerization

**Figure 2:**
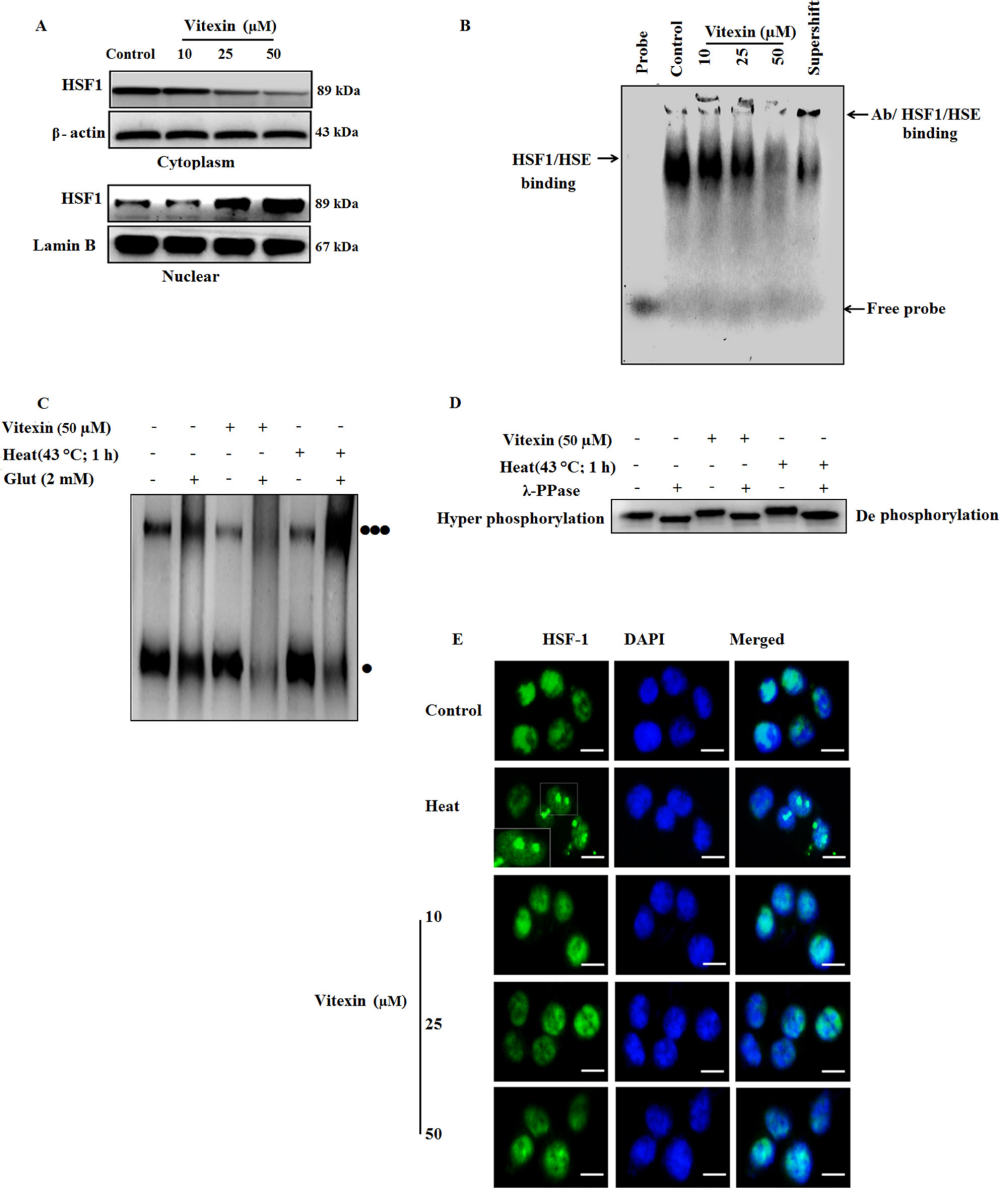
Vitexin alters the oligomeric status of HSF-1 and affects HSE DNA binding (**A**) HCT-116 cells were treated various concentrations of vitexin for 24 h. Cytosolic and nuclear fractions were isolated and run by western blotting probed with antibody to HSF-1. (**B**) EMSA assay was performed to test HSE DNA binding activity using probe HSE consensus oligonucleotides. (**C**) HSF-1 oligomerization was performed by treating aliquots of protein with glutaraldehyde (2 mM) and the reaction was stopped by 1 M lysine. Lysates were run on 10% SDS-PAGE and proteins were probed with anti-HSF-1 antibody [Monomer: 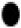] [Trimer: 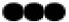] (**D**) Cell lysates were incubated with or without λ-phosphatase for 30 min and run on 10% SDS-PAGE, and probed with HSF-1 antibody, to demonstrate HSF-1 phosphorylation. (**E**) Intracellular localization of HSF-1 in HCT-116 cells exposed to various concentrations of vitexin and stained with anti-HSF-1 antibody. HSF-1 nuclear granules are formed upon heat shock in HCT-116 cells. Scale bar = 50 μm. The data represents mean ± SD of three independent experiments, *n* = 3.

In context with previous findings (Figure 1), chemotherapy drugs result in HSF-1 translocation from cytosol to nucleus, which was evidenced by vitexin treatment in nuclear extracts (Figure 2A; Supplementary Figure 3B). EMSA analysis shows increased HSF-1 DNA binding activity in unstressed cells due to the prominent nuclear localization of HSF-1 in carcinoma cells [6]. Despite of increased nuclear HSF-1 expression (Figure 2A; Supplementary Figure 3B), surprisingly, HSF-1 DNA binding is reduced by vitexin treatment (Figure 2B; Supplementary Figure 4A), which incites us to explore the transactivation steps of HSF-1 which are hampered by vitexin.

The above results prompt us to investigate two main steps of HSF-1 activation mainly, HSF-1 oligomerization and hyperphosphorylation status by vitexin treatment. For HSF-1 oligomerization, we checked the effect of vitexin-treated HCT-116 cell lysates in the presence of glutaraldehyde. Control and heat-shock lysates showed evidence of oligomerization when treated with cross-linker glutaraldehyde [three black dots] (Figure 2C, Supplementary Figure 4B). Vitexin produced an apparent alteration in HSF-1 mobility and inhibits HSF-1 oligomerization in response to glutaraldehyde, with highly pronounced monomeric bands [one black dot] (Figure 2C, Supplementary Figure 4B). As HSF-1 hyperphosphorylation proceeds with oligomerization step, therefore, we checked whether inhibition of HSF-1 oligomerization exerts same effect on the levels of HSF-1 phosphorylation. Surprisingly, vitexin caused HSF-1 hyperphosphorylation and was observed to be down-shifted in the presence of phosphatase. This observation was found similar to heat shocked cells, which suggest that vitexin do not suppresses HSF-1 phosphorylation (Figure 2D; Supplementary Figure 4C). These oddities in results raises another query that inspite of inhibition of oligomerization, what causes the hyperphosphorylation of HSF-1 by vitexin treatment. HSF-1 immunofluorescence shows that vitexin results in increased diffuse nuclear distribution of HSF-1 protein, in contrast with evident well-localized, intensely stained stress granules which are transcriptionally active in heat-treated cells [25] (Figure 2E). These results showed that vitexin caused transcriptional inactivation of HSF-1 that resulted in down-regulation of Hsps synthesis.

### Vitexin suppresses HSF-1-DNA interaction

**Figure 3:**
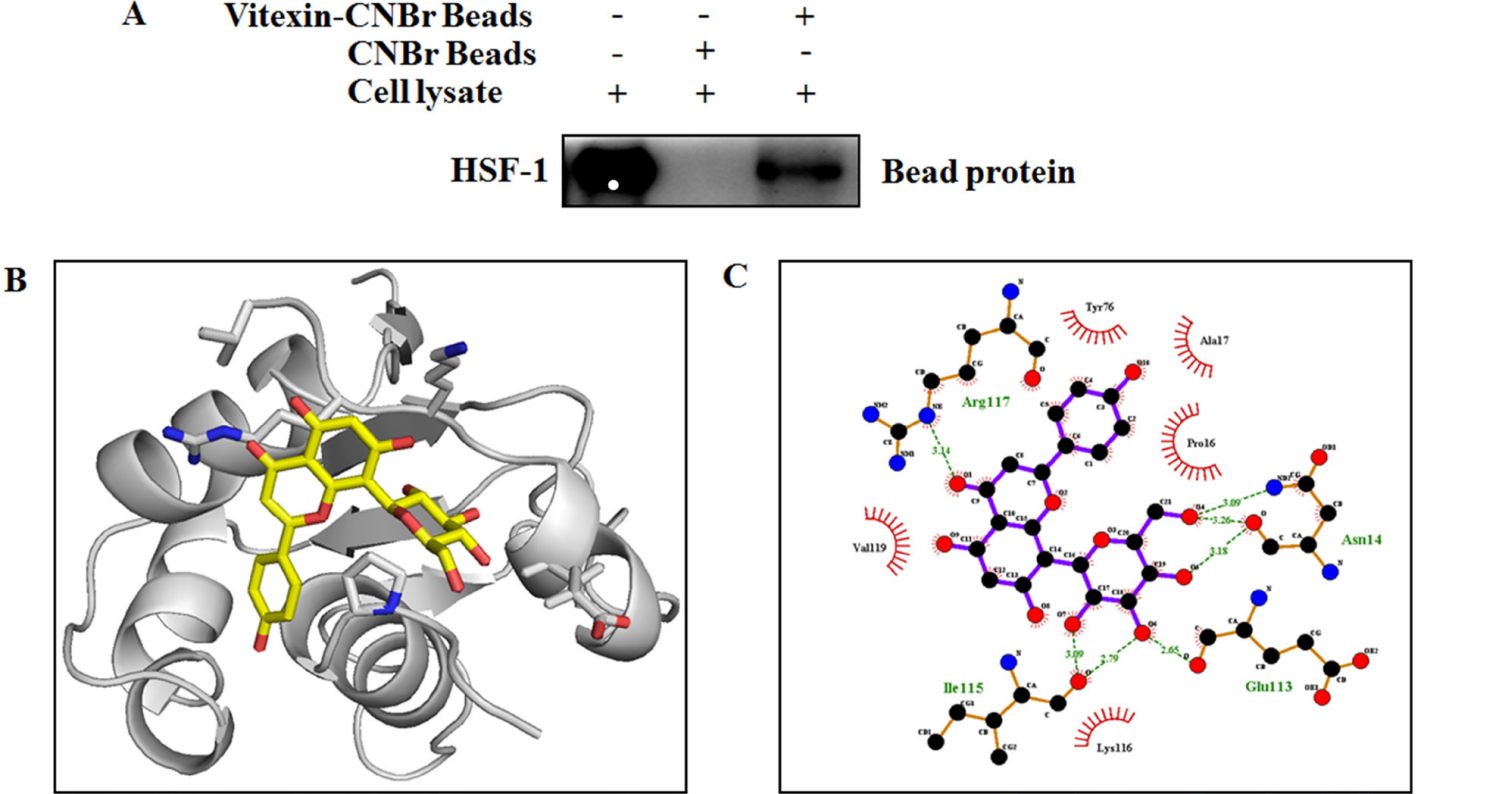
Vitexin binds to the DNA binding domain of HSF-1 (**A**) Vitexin directly binds with HSF-1 in HCT-116 colorectal cancer cells. The binding of vitexin with HSF-1 in HCT-116 was detected by immunoblotting with HSF-1 antibody: lane 1 (input control), whole-cell lysates from HCT-116 cells; lane 2 (control) lysates from HCT-116 cells; and lane 3, whole-cell lysates from HCT-116 cells precipitated with Vitexin-Sepharose 4B beads. (**B**) An interaction between vitexin and HSF-1 is shown in docked complex. Vitexin is shown in stick model and HSF-1 is represented in ribbon model. (**C**) LigPlot analysis of docked vitexin with HSF-1, where hydrogen bonding is represented as green line (- - - -) and hydrophobic interaction is represented as (
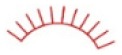
).

Vitexin hampers HSF-1-DNA binding (Figure 2), which prompted us to assess whether HSF-1 could be a potential target of vitexin. HSF-1 was pulled down by vitexin-CNBr beads as compared to CNBr beads alone (Figure 3A), which shows that HSF-1 is the target candidate of vitexin. Furthermore, we approached *in silico* analysis to identify the interaction and binding sites between HSF-1 and vitexin that caused disruption of HSF-1-DNA interaction. Human HSF-1 consists of N-terminal DNA-binding domain (DBD), followed by coiled-coiled oligomerization domain (HR-A/B), a variable regulatory domain (RegD) and a transactivation domain (TAD) [26]. RegD and TAD are highly unstructured due to (a) low abundance of secondary and tertiary structures, (b) absence of hydrophobic core and (c) high structure flexibility, which makes this region unsuitable for docking [27]. With the disparities in HSF-1 structural organization, we targeted HSF-1 DBD region as the suitable target for molecular docking with vitexin. Docking of HSF-1 with vitexin yielded binding affinity score (ΔG_bind_ = -6.7 kcal/mol) to interpret the best conformation. Hydrogen (H) bonding plays a crucial role for inhibition function of any biological molecule [28]. Computational model of vitexin-HSF-1 complex (Figure 3B), were stabilized by three H-bonds (Asn14), two H-bonds (Ile115) and one H-bond (Glu113 and Arg117) residues of HSF-1 respectively (Figure 3C). Hydrophobic interactions with Pro16, Ala17, Tyr76, Lys116 and Val119 strengthen the interaction of vitexin with HSF-1 DBD region (Figure 3C).

### JNK phosphorylates HSF-1

**Figure 4:**
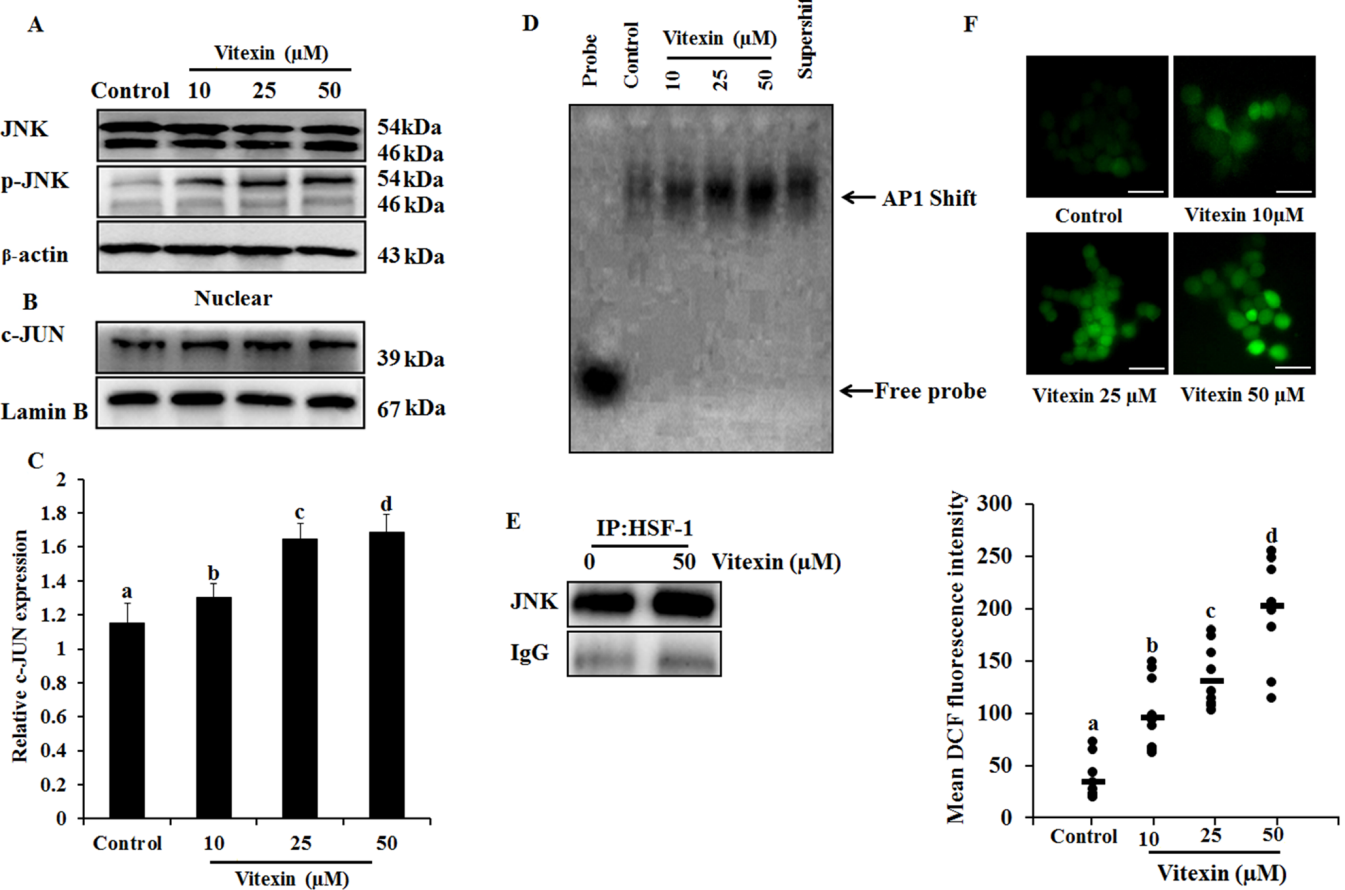
JNK activation and ROS generation by vitexin (**A**) Cells were treated with various concentrations of vitexin for 24 h. Levels of total JNK and p-JNK were determined by western blot. (**B**) Nuclear extract was prepared after vitexin treatment to test c-JUN expression. (**C**) Corresponding quantification of c-JUN blot performed by Image J. (**D**) AP1 DNA binding activity was determined by EMSA analysis using biotinylated consensus AP-1 specific oligonucleotide. (**E**) HCT-116 cells were treated with vitexin and cell lysates were immunoprecipitated using anti-HSF-1 antibody. The precipitated proteins were subjected to SDS-PAGE and western blot analysis using anti-JNK antibody. (**F**) Cells were loaded with H_2_DCFDA for 30 mins and ROS levels were determined by fluorescence microscopy. Representative images and quantitative analysis of ROS generation were determined by ImageJ. Scale bar = 0.1 mm. The data represents mean ± SD of three independent experiments, *n* = 3. Values with different letters (a-d) differ significantly from each other (*p* < 0.05).

The anomaly of observations (Figure 2C, 2D) is in apparent conflict where changes in HSF-1 phosphorylation comply with HSF-1 oligomerization [29]. HSF-1 transactivation is depended on its distinct phosphorylation sites [30], and c-Jun NH_2_ terminal kinase (JNK) phosphorylates HSF-1 at Ser^363^ which inhibits HSF-1 transcriptional activation [31]. Increase in JNK activity (Figure 4A; Supplementary Figure 5A) by vitexin promoted increased nuclear expression of c-JUN (Figure 4B, 4C) and promotes c-JUN AP1 DNA binding in dose-dependent manner, which represses HSF-1 activity (Figure 4D; Supplementary Figure 5B). In light of the observation of JNK-mediated HSF-1 phosphorylation, we performed immunoprecipitation analysis to determine whether these two proteins physically associate. Immunoblot analysis of HSF-1 immunoprecipitates shows the presence of increased JNK by vitexin treatment (Figure 4E; Supplementary Figure 5C). These results showed that JNK activation by vitexin lead to hyperphosphorylation and inactivation of HSF-1 activity. Considerable evidence has delineated the upstream pathway of ROS generation mediated sustained JNK activation [32]. Vitexin (10 μM) initiated ROS generation which was explosively increased in 25 and 50 μM concentrations (Figure 4F), suggesting that vitexin promoted JNK mediated ROS induction. In order to determine whether JNK activation leads to ROS induction, we analyzed ROS levels in the presence of JNK inhibitor SP600125 by H_2_DCFDA assay. We observed that SP600125 neutralizes ROS generation that was induced by vitexin treatment in HCT-116 cells for 24 h (Supplementary Figure 6). These results showed that vitexin caused JNK-mediated ROS induction in HCT-116 cells.

### Vitexin promotes autophagy induction

**Figure 5:**
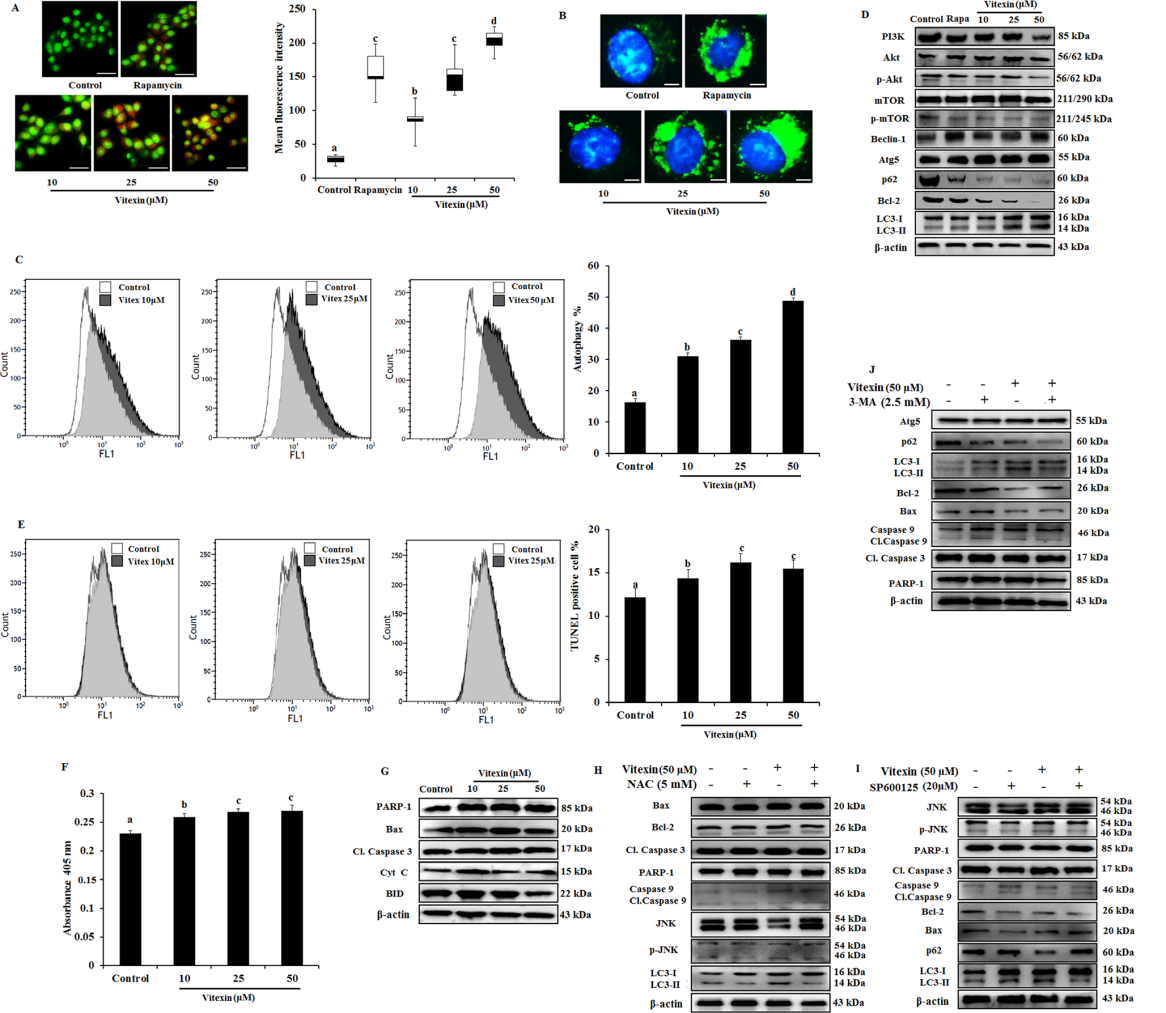
Evidence that vitexin induces autophagy which contributes to cell death (**A**) Cells treated with or without vitexin and rapamycin, and stained with acridine orange to determine autophagosome formation (bright orange granules), using fluorescence microscope (scale bar = 0.1 mm). Quantitative analysis of autophagosome formation was shown. (**B**) Autophagic vesicles (green) stained with Cyto-ID Green autophagy dye and nuclei counterstained with Hoechst 33342 (blue) were observed by fluorescence microscopy (scale bar = 50 μm). (**C**) Flow cytometry was performed to analyze cell autophagy with Cyto-ID green autophagy reagent. (**D**) Immunoblot analysis for autophagy marker proteins was performed using protein homogenates from HCT-116 cells. (**E**) Cell apoptosis was analyzed by TUNEL assay flow cytometry (**F**) Apostrand ELISA apoptosis assay after treatment with vitexin for 24 h. (**G**) The expression of apoptosis markers was determined by western blot. Cells were preincubated with (**H**) NAC (5 mM; 12 h), (**I**) SP600125 (20 μM; 2 h) or (**J**) 3-MA (2.5 mM; 1 h) and then treated with vitexin (50 μM; 24 h). The expressions of apoptosis and autophagy proteins were measured by western blot. The data represents mean ± SD of three independent experiments, *n* = 3. Values with different letters (a-d) differ significantly from each other (*p* < 0.05).

HSF-1 is reported to promote cancer cell survival and enhanced chemotherapy resistance through autophagy [33]. However, overwhelming evidences suggested that ROS mediated JNK activation promote both apoptotic and autophagy mediated cell death [34]. To evaluate whether vitexin caused type I (apoptosis) or type II (autophagy) programmed cell death, we analyzed both cellular death pathways in vitexin-treated HCT-116 cells. Formation of acidic compartments was visualized with acridine orange staining; a weak base that accumulates in acidic spaces and fluoresce bright orange. The autophagy inducer rapamycin was used as positive control which promoted development of brighter orange fluorescence in HCT-116 cells. Vitexin causes elevated brighter orange fluorescence, and promoted a substantial increase in autophagosomes and autophagic flux in HCT-116 cells as observed by Cyto-ID and flow cytometry respectively in a dose-dependent manner (Figure 5A–5C). Phosphatidylinositol 3-kinase (PI3K)/Akt/mammalian target of rapamycin (mTOR) pathway is crucial intracellular signaling pathway in regulating cell growth, tumorigenesis and autophagy induction [35]. Immunoblot analysis showed that vitexin down regulated PI3K, p-Akt, p-mTOR, p62, a polyubiquitin-binding protein and autophagy repressor protein Bcl-2 expression and increased the expression of autophagy marker proteins Beclin-1, Atg5 and microtubule-associated protein light chain 3 (LC3)-I conversion to lipidated form LC3-II, which promotes autophagy induction (Figure 5D, Supplementary Figure 7A, 7B). Rapamycin caused autophagy induction by inhibiting p-mTOR expression and upregulates autophagy marker protein levels (Figure 5D, Supplementary Figure 7A, 7B).

Apart from autophagy generation, we examine at molecular levels whether vitexin could induce apoptosis. Cellular apoptotic induction by flow cytometry using Terminal deoxynucleotidyl transferase dUTP nick end labeling (TUNEL) assay and ApoStrand^™^ Apoptosis Detection Kit, suggests that vitexin slightly induced the apoptosis pathway (Figure 5E, 5F). Western blotting analysis reveals the expression levels of pro-apoptotic marker proteins like Bax, Cytochrome c, BID PARP and caspase-3 were found to be reduced or no effect by vitexin treatment (Figure 5G; Supplementary Figure 8). Our findings were validated by using NAC (ROS scavanger), SP600125 (JNK inhibitor) and 3-MA (autophagy inhibitor) that mitigates autophagy and did not promote apoptosis induction (Figure 5H–5J). These results showed that vitexin promotes caspase-independent cell death in HCT-116 cells.

### Apolipoprotein L1 (ApoL1) is essential for autophagic cell death

**Figure 6:**
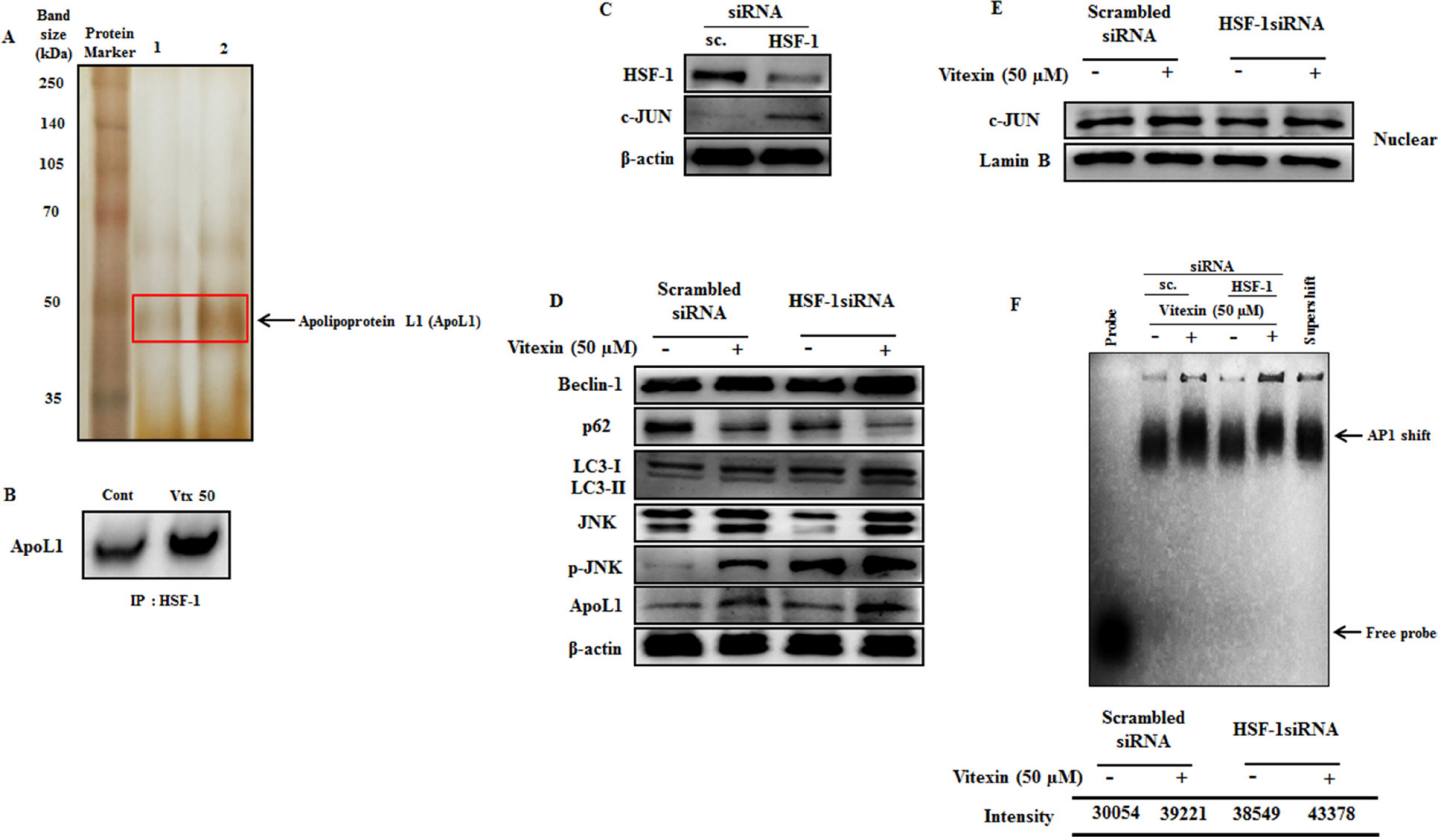
Vitexin promotes induction of JNK and BH3-only protein ApoL1 in HSF-1 knockdown cells (**A**) Polypeptide profiling of HSF-1 immunoprecipitated product in HCT-116 control cells (Lane 1) and treated with 50 μM vitexin (Lane 2), identified apolipoprotein L1 (ApoL1) as a novel marker for autophagic cell death. Samples were loaded on 10% SDS-PAGE and were silver stained. Protein bands were cut out and analyzed by MALDI mass spectroscopy. (**B**) The HSF-1 precipitated proteins were subjected to SDS-PAGE and western blot analysis using anti-ApoL1 antibody. (**C**) Cells were targeted with non-tageting (scrambled) siRNA or HSF-1 siRNA. Cell extracts were subjected to western blotting using antibodies against HSF-1, c-JUN and β-actin. (**D**) Scrambled and HSF-1 transfected cells were treated with vitexin (50 μM) and relative protein expression level was analyzed using Western blotting. Nuclear extracts was prepared to analyze (**E**) c-JUN expression and (**F**) AP1 DNA binding affinity by EMSA assay. The data represents mean ± SD of three independent experiments, *n* = 3.

According to the above results (Figure 5), we prompt to investigate the possible molecular basis for caspase independent autophagy cell death exerted by vitexin after HSF-1 inhibition. Previous evidences suggest that Bcl-2 family members play crucial roles in determining interplay between autophagic and apoptotic cell death [36]. To determine whether Bcl-2 family proteins get activated after vitexin treatment, we performed MALDI-TOF analysis of HSF-1 immunoprecipitates in both absence and presence of vitexin. Interestingly, MALDI-TOF mass spectrometry and immunoblot analysis of HSF-1 immunoprecipitates identified increased expression of ApoL1 in vitexin treated HCT-116 cells (Figure 6A, 6B; Supplementary Figure 9A). The obtained result suggests that ApoL1 could be a novel interacting protein of HSF-1, which drives the autophagy cell death program in the presence of vitexin. ApoL1, a novel Bcl-2 homology domain 3 (BH3)-only pro-death protein, when accumulated and overexpressed intracellularly contributes to autophagic cell death by formation of the AVOs, accumulation and translocation of LC3-II [16]. To further validate our results whether vitexin promotes JNK and ApoL1 mediated cell death by autophagy, we check the effect of vitexin in HSF-1 knock down HCT-116 cells. Silencing of HSF-1 expression using siRNA caused a marked increase in c-JUN protein levels, deciphering HSF-1-JNK interaction (Figure 6C; Supplementary Figure 9B). Increased expression of ApoL1 and autophagy induction was detected, as evidenced by increased p-JNK, Beclin-1 and LC3-II expression levels after vitexin treatment (Figure 6D; Supplementary Figure 10A). Enhanced nuclear expression of c-JUN (Figure 6E; Supplementary Figure 10B) and EMSA analysis showed strong AP1 DNA binding after vitexin treatment (Figure 6F). This result demonstrates that HSF-1 knock down promotes JNK signaling with concomitant expression of ApoL1 which contributes to autophagic mediated cell death.

### Vitexin inhibits *in vivo* growth of colorectal carcinoma

**Figure 7:**
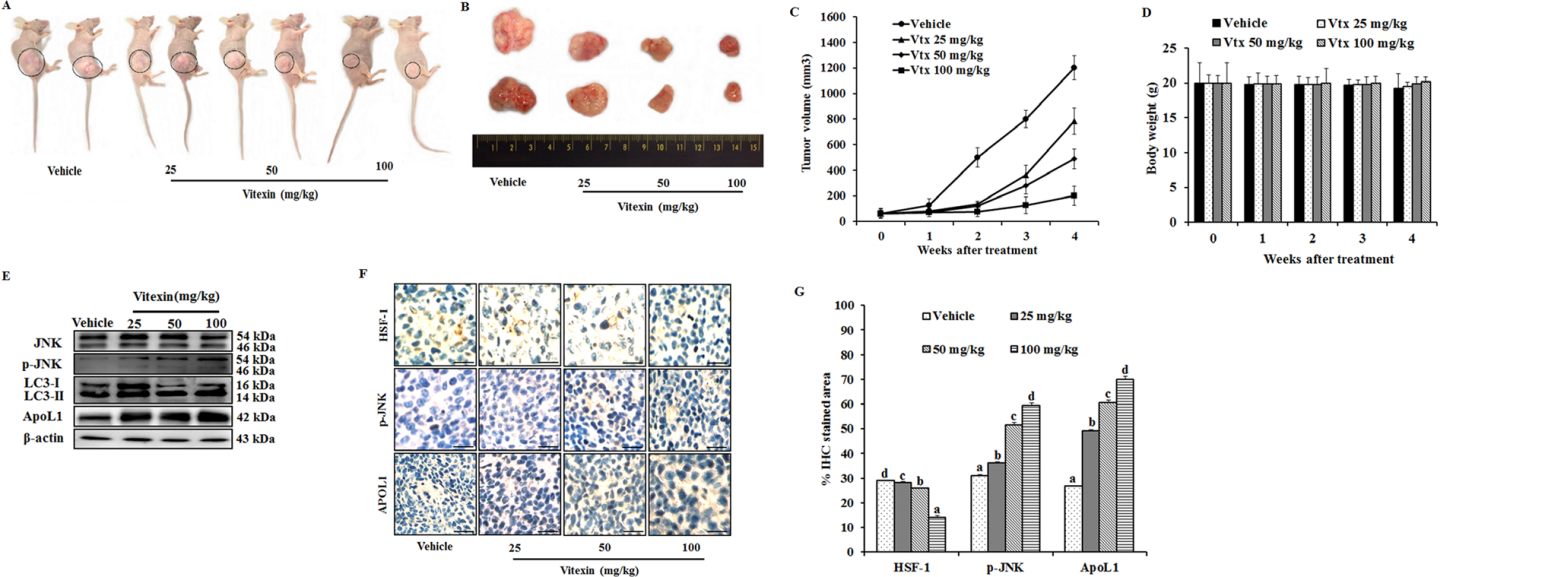
Vitexin inhibits growth of human colorectal xenograft *in vivo* Mice were inoculated subcutaneously in the right flank with HCT-116 cells. (**A**) Representative images of HCT-116 xenograft nude mice showing regression in tumor volume after treatment with vitexin in comparison to vehicle treated group. (**B**) Dissected out tumors from vehicle treated and vitexin treated xenograft mice. Change in (**C**) tumor volume (**D**) and body weight of the mice were observed after initiation of treatment. (**E**) Representative blots are shown to analyze total JNK, p-JNK, LC3-I/II and ApoL1 in vehicle and vitexin treated xenograft tissues. (**F**) The expression levels of HSF-1, p-JNK and ApoL1 were also examined by immunohistochemistry (scale bar = 0.1 mm). (**G**) Percentage of IHC stained area or representative proteins were quantified by ImageJ. The data represents mean ± SD of three independent experiments, *n* = 6. Values with different letters (a-d) differ significantly from each other (*p* < 0.05).

Since we observed an inhibition of cell viability by vitexin *in vitro*, we evaluated whether these observations could be deciphered in animal model system. To determine the *in vivo* effect of vitexin on colorectal cancer, vitexin was orally administered at doses of 25, 50 and 100 mg/kg in mice xenograft model. As shown in Figure 7A–7C, vitexin treatment resulted in a marked decline of HCT-116 xenograft tumor growth and tumor volume dose-dependently. Notably, the body weight of mice from vitexin treated groups did not significantly differ from the vehicle control following 4 weeks of drug exposure (Figure 7D), suggesting that vitexin has no severe toxicity to the mice. Western blot analysis from tumor homogenates show that vitexin increased in levels of p-JNK, LC3-II and ApoL1 (Figure 7E).

HSF-1 is associated with tissue-specific tumorigenesis in a number of mouse models, and has been used as a prognostic marker of cancer types. HSF-1 has been found to be associated with several oncogenic pathways that have important roles in maintaining tumor development. HSF-1 is able to associate with cell cycle regulators and modulate the metabolism of glucose and lipids by indirectly regulating insulin receptor protein expression, which has been demonstrated to be important for tumor initiation and development [37, 38]. Therefore, colorectal cancer stage progression is likely to be correlated with the expression of HSF-1, which indicates that HSF-1 may be a useful biomarker for CRC prognosis and the development of novel therapeutic strategies for its treatment. Immunohistochemistry demonstrated increase in percentage of IHC stained area for p-JNK and ApoL1, whereas a decrease in HSF-1 stained percentage area was observed (Figure 7F, 7G). These *in vivo* results revealed that vitexin inhibits the growth of colorectal carcinoma with low toxicity levels.

## DISCUSSION

As a master regulator of HSR, HSF-1 enhances organism survival during environmental challenges. Contrastingly, it also supports malignant transformations by enabling the cells to combat with inevitable drastic alterations of intracellular milieu, which could be fatal to host. The repertoire of HSF-1-regulated genes in cancer extends far beyond protein folding, which includes from energy metabolism to extracellular matrix formation. As a hallmark for cancer, proteomic instability has received less attention when compared with the widely recognized genomic instability [1]. Vitexin is a derivative of apigenin flavonoid belonging to the flavone structural class. Till date, the probable mechanistic action of anti-cancer activity of vitexin against colorectal cancer in the context of HSF-1 as probable target molecule has not been reported yet. In our present study, we explored the effect of vitexin on several proteins and signaling pathways to determine how HSF-1 and autophagy are linked. We have demonstrated that vitexin down regulates the expression levels of key molecular chaperones which contribute for reduced cell viability (Figure 1). We hypothesized that vitexin treatment may exert an effect on HSR by modulating the complex activation process of a negatively regulated key transcription factor known as HSF-1. Intermediate forms of HSF-1 (monomeric and dimeric) can bind to DNA consensus sequence, but the trimeric form is believed to be required for full activation of its transcription [39].

We observed that vitexin causes a profound increase in nuclear expression of HSF-1 in a diffused pattern, in contrast to characteristic stress granules formation during heat stress as observed in immunofluorescence which represents transcriptionally inactive HSF-1 trimers (Figure 2E). HSE DNA binding assays revealed that DNA binding disappeared in the presence of vitexin (Figure 2B). Cross-linking and phosphorylation studies revealed that vitexin treatment inhibited the formation of HSF-1 oligomerization, whereas, hyperphosphorylation of HSF-1 was found evident (Figure 2C, 2D). However, HSF-1 phosphorylation was found to be less prominent as compared to heat-shocked cells. The possible explanations for this anomaly are: In early response to a stressful event, trimerization of HSF-1 may occur simultaneously or may even precede the induced phosphorylation of HSF-1 [40]. One or both regulatory events of HSF-1 transactivation function may be modulated by phosphorylation of certain sites in the factor itself [41]. A similar kind of mechanism was observed by treatment of high dosage of calyculin A (20 nm), a protein phosphatase inhibitor, hyperphosphorylated the HSF-1 prior to heat shock and retarded HSF-1 trimer formation [42]. Additionally, EMSA analysis does not detect a change in phosphorylation of single residue rather than, it provides information on overall changes in HSF-1 phosphorylation.

Interaction between vitexin and DBD of HSF-1 speculated an inhibitory effect of vitexin against HSF-1. Structural data analysis of HSF-1 DBD demonstrated that conformation of C-terminal residues (101–120) of human HSF-1 DBD are stabilized by hydrophobic interactions of Ile115 and other residues, which allows DBDs and coiled-coil bundle to locate on opposite faces of DNA double helix, thus facilitating HSF-1 trimer to wrap around DNA. Additionally, conserved residues including Tyr76 stabilizes and orient helix in the major groove by forming hydrogen bonds to the phosphate backbone. Arg117 formed additional contacts across the major groove, whereas, Lys116 is solvent exposed and prone to modification by acetylation [26]. These contacts between DBDs would facilitate dynamic contacts of HSF-1 DNA complex. We speculate by docking and Ligplot analysis (Figure 3B, 3C) that vitexin form hydrogen bonding and hydrophobic interactions with these residues, which may have caused conformational changes, that may have affected HSF1 activity and hindered the orientation of coiled-coil oligomerization domain.

Previous reports suggested that cells overexpressing JNK showed a rapid disappearance of HSF-1 granules resulting in suppression of its transcriptional activity [41]. Congruent with our findings, cisplatin treatment in A549 cells hampers HSF-1 DNA by preventing HSF-1 trimerization and JNK induced HSF-1 phosphorylation [41]. Under physiological growth conditions JNK remains less active, and is highly sustainable upon encountering stress. This cause rapid repression of HSF-1 transcriptional activation and prevent Hsps accumulation, due to which misfolded proteins could not be repaired and causes cell death [31]. Vitexin results in enhanced JNK activity which causes rapid loss of HSF-1 DNA binding activity (Figure 2B) and reduction in Hsps production (Figure 1E, 1F). IP data (Figure 4E) suggest that vitexin enhanced JNK binding to HSF-1 that causes JNK activation (Figure 4B–4D), which phosphorylates and suppresses transcriptional activation of HSF-1.

Autophagy has been under extensive investigation with its implication of its dual role (cell survival or cell death) for therapeutic purposes in cancer. Considerable evidences have delineated the complex ROS-JNK pathway induced by natural compounds which promote autophagic cell death that can be effective in human cancer treatment [42, 43]. Excessive ROS generation transduces JNK phosphorylation in response to stress signals mediated by HSF-1 inactivation which damage cellular components leading to cell apoptosis and autophagy [44]. In the current study, vitexin induced marked increase in ROS generation (Figure 4F) that causes JNK activation (Figure 4A) and autophagy induction (Figure 5A–5D). Accumulation of acidic vesicles, autophagosome formation by Cyto ID fluorescence, flow cytometry, upregulation of autophagy marker proteins and subsequent decrease in PI3K/mTOR/Akt pathway showed that vitexin results in autophagy induction. Parallerly, vitexin does not promote apoptosis as demonstrated by reduced absorbance (apoptosis assay) with no substantial changes in pro-apoptotic protein levels. (Figure 5E–5G), which indicates that vitexin does not support apoptotic cell death. Our results were further confirmed by ROS inhibitor, NAC and JNK inhibitor, SP600125 which shows no considerable changes in apoptosis induction, whereas JNK phosphorylation was abolished and autophagy induction was abrogated (Figure 5H–5I). Interestingly, autophagy inhibitor 3-MA, does not support apoptosis induction in parallel to p-JNK attenuation (Figure 5J), indicating that vitexin contributed to autophagy-mediated cell death.

A complex relationship exists between autophagy and apoptosis, and evidences suggest their interplay for cooperation, assistance and antagonizing effect to each other in cancer cell death. In our study, we did not observe significant levels of apoptosis induction in the presence of NAC, SP600125 and 3-MA along with vitexin, which instigated to examine the probable driving factor behind HSF-1-mediated non-caspase-dependent ACD. In an effort to discern novel protein and its probable interaction with HSF-1 in ACD, we recognize a Bcl-2 family protein ApoL1. ApoL1, a *bona fide* BH3-only pro-death protein, is primate-specific and has been reported to be relevant for mediating cytotoxicity via autophagic cell death in cultured podocytes [45]. Additionally, ApoL1 expression leads to JNK hyperactivation and other MAPKs act as mediators of ApoL1 nephrotoxicity [46]. ApoL1 mediated ACD is characterized by accumulation and translocation of LC3-II, formation of acidic vacuoles, restraint apoptogenic factors and caspase suppression [16]. Interestingly, MALDI-TOF and immunoblot analysis revealed an increase of ApoL1 protein in vitexin treated HSF-1 immunoprecipitates (Figure 6A, 6B), implying the fact that ApoL1 assist in promoting ACD. In context of autophagy, p62/SQSTM1 has been identified as an HSF-1 dependent gene [47]. We used siRNA to silence HSF-1 and then subsequently treated cells with vitexin. In HSF-1 silenced cells, c-JUN was found to be highly expressed and was mainly translocated to nucleus, with strong AP1 DNA binding (Figure 6C, and 6F). Our data was further validated by strong induction of p62 in scrambled siRNA, whereas, low or absent in HSF-1 silenced cells after vitexin treatment. JNK phosphorylation followed by Beclin-1 induction, increased LC3-II turnover and enhanced ApoL1 expression was observed in HSF-1 silenced cells with vitexin treatment (Figure 6E).

In our *in vivo* study, we found that vitexin inhibited tumor growth (Figure 7A–7C). Western blot and immunohistochemical analysis confirmed the decrease in HSF-1 levels, with increase in p-JNK, LC3-II and ApoL1 levels following vitexin treatment (Figure 7E, 7F). It is noteworthy that vitexin caused no considerable changes in body weight of mice (Figure 7D), revealing that vitexin exerts its therapeutic efficacy against colorectal xenograft with minimal side effects.

Previous studies showed that vitexin has antioxidant properties, attenuates heat stress induced oxidative stress in A549 cells [48–50] and cause apoptotic cell death in various cell lines [51, 52]. These findings conflict with our current results that vitexin induces ROS generation in colorectal cells. The discrepancy of these findings may be attributed to the difference in cell lines and time duration of dose treatment. We can speculate the effect of vitexin on oxidative stress is likely to be dependent on cell type and time duration of dose treatment.

To summarize, our study is the first to demonstrate that vitexin can effectively inhibit the proliferation of colorectal carcinoma cells by inhibiting HSF-1 activity, and lead to cell death by inducing autophagy mediated by JNK and ApoL1 activation. In addition, we determined that vitexin binds to DNA binding domain of HSF-1, inhibits HSF-1 oligomerization and blocked HSF-1 DNA binding. A physical association of JNK and ApoL1 with HSF-1 was established and their activation further assists in mediating ACD by vitexin treatment. In the xenograft model, vitexin also shows marked antitumor activity with low levels of toxicity. These compelling evidences expand our understanding by revealing the importance of HSF-1 in autophagy regulation, which have likely significance in cancer research.

## MATERIALS AND METHODS

### Reagents

Vitexin, acridine orange (AO), Cyanogen bromide (CNBr) sepharose 4B beads, 3-(4,5-dimethyl-2-thiazolyl)-2,5-diphenyl-2H-tetrazolium bromide (MTT), dichlorodihydrofluorescein diacetate (H_2_DCFDA), N-acetyl-l-cysteine (NAC), SP600125, 3-methyladenine (3-MA), dimethylsulfoxide (DMSO), rapamycin (rap) and other chemicals and pure drugs were of reagent grade and were purchased from Sigma-Aldrich (St. Louis, MO, USA). For details, see Supplementary Tables 1, 2.

### Cell culture and compound treatment

HCT-116 colon cancer (ATCC, Rockville, MD) were cultivated in RPMI 1640 supplemented with 10% fetal bovine serum (Gibco BRL, Gaithersburg, MD), 25 mM HEPES buffer, 1% Pen-Strep Cocktail (Sigma, St. Louis, MO, USA) and maintained at 37°C (95% air, 5% CO_2_). Normal colon cell line CCD-112-CoN fibroblast (ATCC, Rockville, MD) was cultivated as per ATCC guidelines. After attaining 80–90% confluency (1 × 10^5^ cells/well), cells were treated with vitexin (5% DMSO) at concentration for 10, 25, and 50 μM for 24 h for further experimental analysis.

### Vitexin-Sepharose 4B beads preparation and *ex-vivo* pull-down assay

Vitexin can be easily coupled with sepaharose beads to yield vitexin-CNBr-activated Sepharose 4B complex. For the *ex vivo* pull-down assay, a total of 500 μg of HCT-116 protein extract was incubated with 50% slurry of vitexin-CNBr or Sepharose 4B beads (as a negative control) in reaction buffer.

### Molecular docking

3D structure of HSF-1 (PDB ID: 5D5U) was available at Protein Data Bank (www.rcsb.org) which was retrieved in PDB format. Structure of vitexin (ZINC04245684) was retrieved from ZINC database [53]. Molecular docking was carried out using Autodock Vina [54], which uses pdbqt input files both for ligands as well as protein, to study the nature of interaction of HSF-1 with vitexin, An energy grid box of center grid coordinates used in this study, for Autodock Vina were -10.322 × 4.672 × 23.12 while size used for x, y, z were 44 × 28 × 36, respectively with 1.000Å spacing. Protein residues forming hydrogen and hydrophobic interactions with ligand molecule is further calculated by Ligplot+ software [55].

### Morphological findings

HCT-116 cells were cultured and treated with 10, 25 and 50 μM concentration of vitexin. Cell morphology was observed microscopically (20×) 24 h after addition (Nikon Eclipse TS100, Epi-fluorescence microscope, Nikon Corp., Tokyo, Japan).

### Determination of cytotoxicity and plasma membrane integrity

After vitexin treatment for 24 h, cell cytotoxicity was measured as optical density at 540 nm with ELISA reader (Bio-Tek Instrument Co., WA, USA) 4 h after the addition of MTT working solution (5 mg/ml). Plasma membrane integrity was assessed on lactate dehydrogenase (LDH) leakage into the culture medium from cells using LDH assay kit (Sigma-Aldrich, St. Louis, MO, USA) as per manufacturer's instructions.

### Gel mobility shift assays

Electrophoretic mobility shift assay (EMSA) analyses were performed using a LightShift^®^ Chemiluminescent EMSA Kit (Thermo Scientific). Nuclear extracts (1 μg each) were examined for their binding capacity to 20 fmol of the biotin-labeled standard HSE and activator protein 1 (AP1) binding motif (Supplementary Table 3). The reaction in the supershift lane contains a selective antibody which causes further retardation within the gel when bound to the protein–DNA complexes. Binding reaction and electrophoresis were performed on 4% native polyacrylamide gel and complexes were then transferred onto Biodyne^®^ precut nylon membranes (Thermo Scientific). Biotin-labeled DNA was detected by chemiluminescence as recommended by the manufacturer.

### RNA isolation and quantitative real-time PCR analysis

Total RNA content of cells was isolated using a RNA-spin™ Total RNA extraction kit according to the manufacturer's protocol (Intron Biotechnology, Republic of Korea). RNA concentrations were determined using a Qubit^®^ 2.0 Fluorometer RNA assay kit (Life Technologies, USA). cDNA was prepared using a Maxime RT Premix cDNA synthesis kit (Intron Biotechnology, Republic of Korea) according to the manufacturer›s protocol. Quantitative PCR was performed using 0.9 μM each of forward and reverse primers (Supplementary Table 4) with SYBR Green premix (Life technologies, USA) on a real-time PCR system (Agilent Technology QPCR System, CA, USA).

### Preparation of whole cell, cytoplasmic and nuclear extracts

Cells were lysed in RIPA buffer and centrifuged at 12,000 rpm for 15 min 4°C to extract whole cell proteins. Cytoplasmic and nuclear proteins were extracted using an NE-PER nuclear protein extraction kit (Thermo scientific) according to the manufacturer's instructions. Protein concentration was measured by bicinchoninic acid (BCA) assay kit (Sigma-Aldrich, St. Louis, MO, USA).

### Immunoblotting

Equal amounts of whole-cell lysates, cytoplasmic or nuclear proteins were separated by SDS gel electrophoresis, transferred using PVDF membrane (Roche Diagnostics, USA), blocked and probed with various primary antibodies. Appropriate secondary antibodies conjugated to HRP were used for detection using an enhanced chemiluminescence (ECL) system (Amersham, Velizy-Villacoublay, France).

### Oligomerization and phosphorylation of HSF-1

For oligomerization, aliquots of protein (10 μg) were mixed with 2 mM glutaraldehyde and allowed to incubate at room temperature for 30 min. The reaction was stopped by the addition of 5 M lysine. Phosphorylation was performed by incubating the cell lysates in the presence or absence of 400 units of λ-phosphatase (New England Biolabs) for 30 min. Both extracts were then run on 10% continuous gradient SDS-PAGE, followed by immunoblotting to demonstrate oligomerization and phosphorylation status of HSF-1.

### Intracellular reactive oxygen species (ROS) detection

Intracellular ROS generation was detected with fluorescent probe H_2_DCFDA. After incubation with vitexin for indicated concentrations, cells were washed and incubated with H_2_DCFDA for 15 min. ROS is oxidized to form highly fluorescent product 2′,7′-dichlorofluorescein which was analyzed with fluorescence microscope (Nikon Eclipse TS100 Epi-fluorescence microscope, Nikon Corp., Tokyo, Japan).

### Immunofluorescence

Cells were seeded on gelatine-coated cover glass (SPL, Republic of Korea) for 24 h before vitexin treatment. As positive control, HCT-116 cells were subjected to heat shock at 43°C followed by 4 h recovery at 37°C to detect HSF-1 granules. After fixation, cells were permeabilized and blocked with 1% BSA at 37°C for 1 h. Samples were incubated with total anti-HSF1 antibody (Santa Cruz, CA, USA) overnight, washed with PBST and were detected by FITC-conjugated secondary antibody (Santa Cruz, CA, USA). Coverslips were mounted on glass slides with DAPI antifade (Invitrogen, USA) staining solution and viewed using a fluorescence microscope.

### Acridine orange staining for acidic compartment evaluation

Cells (1 × 10^5^) were seeded on coverslips (SPL, Republic of Korea) in a 6-well plate and allowed to adhere overnight. Cells were treated with vitexin (24 h) and 500 nM rapamycin as autophagy stimulator for 12 h. Cells were stained in dark with 100 μg/ml of AO in PBS for 10 min and washed with PBS. Fluorescent micrographs were obtained using a fluorescence microscope at 40× magnification. (Nikon Eclipse TS100 Epi-fluorescence microscope, Nikon Corp., Tokyo, Japan).

### Autophagic vesicles staining

Cells were seeded on coverslips (SPL, Republic of Korea) and treated with vitexin and rapamycin (500 nm). Cells were fixed and stained using the Cyto-ID^TM^ Autophagy Detection Kit (Enzo Life Sciences, Plymouth Meeting, PA) according to the manufacturer's instruction and counterstained with Hoechst 33342. Coverslips were mounted on glass slides with antifade (Invitrogen, USA) staining solution and analyzed using a fluorescence microscope. (Nikon Eclipse TS100 Epi-fluorescence microscope, Nikon Corp., Tokyo, Japan).

### Immunoprecipitation (IP) and protein mass fingerprinting

HSF-1 immunoprecipitation assay was performed using Dynabeads^®^ Co-Immunoprecipitation Kit (Novex, USA). Total cell lystaes were incubated with 5 mg of antibody-coupled magnetic Dynabeads^®^ M-270 Epoxy beads and the HSF-1-pull down products were subjected to 10% denaturing polyacrylamide gel electrophoresis followed by immunoblotting. To identify proteins by peptide mass fingerprinting, protein bands were excised, digested with trypsin (Promega, Madison, WI), mixed with a-cyano-4-hydroxycinnamic acid in 50% acetonitrile/0.1% trifluoroacetic acid, and subjected to MALDI-TOF analysis (Microflex LRF 20, Bruker Daltonics, Billerica, MA, USA). Peak list was generated using Flex Analysis 3.0 followed by protein identification using MASCOT, Matixscience (http://www.matrixscience.com).

### Flow cytometry analysis

Apoptosis induction and development of acidic vesicular organelles, were quantified using *in situ* Cell Death Detection Kit (Roche Applied Sciences, Germany) and Cyto-ID^™^ Autophagy Detection Kit (Enzo LifeSciences, Plymouth Meeting, PA) respectively and analyzed by flow cytometry. Cells were stained for 30 min at 37°C, harvested and green (510–530 nm) fluorescence emission from 1 × 10^4^ cells illuminated with blue (488 nm) excitation light was measured with a fluorescent activated cell sorter (FACS). A total of 50000 events were collected and data analysis was performed by using Kaluza flow cytometry software (Beckman coulter, Fullerton, CA, USA).

### siRNA transfection

siRNA transfection was achieved by using Lipofectamine^®^ RNAiMAX Transfection Reagent (Invitrogen, USA). Cells were seeded into 6-well plates overnight and 25 pm of siRNA against HSF-1 (Supplementary Table 5) in 7.5 μl of Lipofectamine were then added to each well. After 24 h incubation with vitexin, cells were lysed and transfection efficiency was determined by evaluating the expression levels of HSF-1 and β-actin using immunoblotting.

### Athymic nude mice xenograft study

Male BALB/c pathogen free athymic nude mice, (4 weeks old; body weight 20–22 g) were purchased from Orient Bio, Inc. (Seoul, Republic of Korea). Mice were housed in a sterile temperature controlled-room on a 12 h: 12 h light: dark schedule with standard rodent chow diet and water *ad libitum*. The guidelines for animal care and handling were approved by the Institutional Animal Care and Use Committee of Daegu University. For engraftment, 1 × 10^7^ HCT-116 cells were inoculated subcutaneously into the right flank region, and mice were monitored for tumor development. When tumors attained the size of approximately 5 × 5 mm^3^, mice were randomly assigned to four groups and received vehicle (5% DMSO), 25, 50 and 100 mg/kg vitexin, by oral administration for three days per week. Each group consisted of five animals. The tumor volumes were monitored for the duration of experiment and estimated by using the formula [(W)^2^ × L]/2, where W represents the width (shortest tumor diameter) and L represents the length (longest tumor diameter). Body weight of tumor-bearing animals was recorded to determine compound toxicity. Tumors were dissected and stored in liquid nitrogen or fixed in formalin for further analysis.

### Immunohistological analysis

Formalin fixed paraffin-embedded tumor specimens were sectioned (4 μM). After being deparaffinized followed by a hydration process, the slides were treated with 3% H_2_O_2_ to block endogenous peroxidase activity. After antigen retrieval, sections were incubated with HSF-1, p-JNK and ApoL1 antibodies followed by PBS washes. Appropriate secondary antibodies were used for detecting immunoreactivity with DAB substrate (Sigma-Aldrich, St. Louis, MO, USA). Background counterstaining was performed with hematoxylin and slides were analyzed under light microscope (×400). The percentage of IHC stained area in tissue sections were calculated by ImageJ (NIH, USA) software.

### Data analysis and statistical procedures

Statistical analysis was performed with SPSS (Statistical Package for the Social Sciences) 22.0 for windows. Results are expressed as the mean ± standard deviation (SD) of three independent experiments. The data were subjected to one-way analysis of variance (ANOVA) and the significance of difference between samples means was calculated by Duncan's multiple range test (SPSS Inc., Chicago, IL, USA). Differences were considered statistically significant for values of *p* < 0.05.

## SUPPLEMENTARY MATERIALS FIGURES AND TABLES


