# Supplementary material for: Vitexin confers HSF-1 mediated autophagic cell death by activating JNK and ApoL1 in colorectal carcinoma cells
Source: Oncotarget. 2017 Aug 10;8(68):112426–41. doi: 10.18632/oncotarget.20113 (PMC5762521; doi:10.18632/oncotarget.20113)
Supplement: Supplementary file 1 [file oncotarget-08-112426-s001.pdf]

Vitexin confers HSF-1 mediated autophagic cell death by activating JNK and ApoL1 in colorectal carcinoma cells

SUPPLEMENTARY MATERIALS

MATERIALS AND METHODS

Method S1 Clonogenicity assay

HCT-116 (~1,000 cells per well) were seeded into 6-well plates with RPMI-1640 culture medium, maintained at 37 °C (95% air, 5% CO2). After attaining semi-confluency, cells were treated with various concentrations of vitexin for 24 h and further maintained for 14 days to allow colony formation. Colonies were stained with 0.1% crystal violet in 50% methanol and 10% glacial acetic acid.

Method S2 Soft Agar Colony formation

HCT-116 cell colony formation was determined by soft agar assay. Cells (2×10^4) were mixed in RPMI-1640 medium containing 0.35% agarose and varying concentrations of vitexin. Then the cell mixture was added on a layer of 0.6% concentration of bottom agar in 6-well plates and allowed to grow for three weeks at 37°C under 5% CO2. Fresh medium containing the vitexin or vehicle (DMSO) was changed every four days. Colonies of 50 cells or more were counted after three weeks.

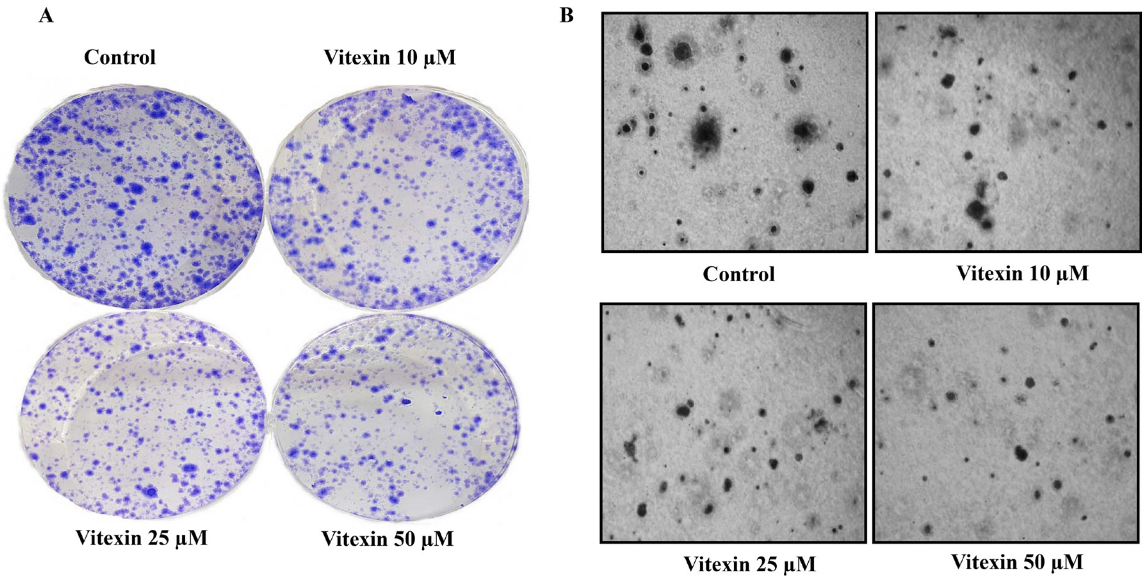

Supplementary Figure 1: The effects of vitexin on HCT-116 cell survival. (**A**) Clonogenic survival assay of HCT-116 cells showed a decreased ability to form colonies following treatment with vitexin for 24 h. (**B**) Anchorage-independent cell growth in soft agar. HCT-116 cells were grown in soft agar for 21 days in the presence of the indicated concentrations of vitexin respectively (40×). The data are represented as mean ± SD of three independent experiments, *n* = 3.

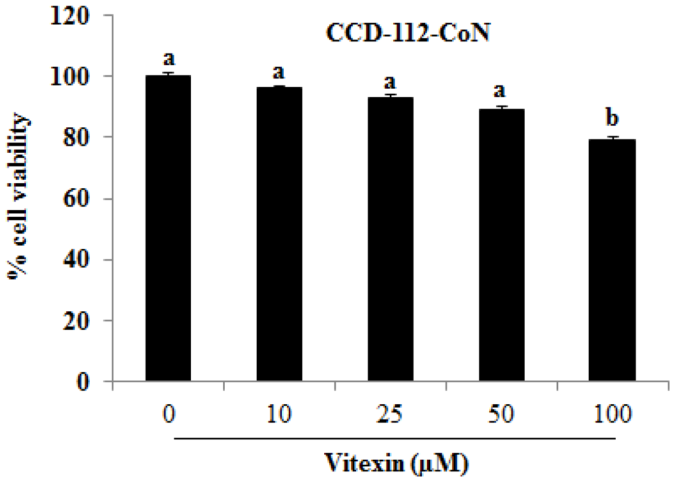

Supplementary Figure 2: Cytotoxicity of vitexin (0-100 µM) for 24 h was determined by normal colon cell line CCD-112-CoN. Normal colon cell line CCD-112-CoN fibroblast were cultivated in DMEM supplemented with supplemented with 10% fetal bovine serum (Gibco BRL, Gaithersburg, MD), 1% Pen-Strep Cocktail (Sigma, St. Louis, MO, USA) and maintained at 37°C (95% air, 5% CO2). The data are represented as mean ± SD of three independent experiments, $n = 3$. Values with letters (a-b) differ significantly from each other ($p < 0.05$).

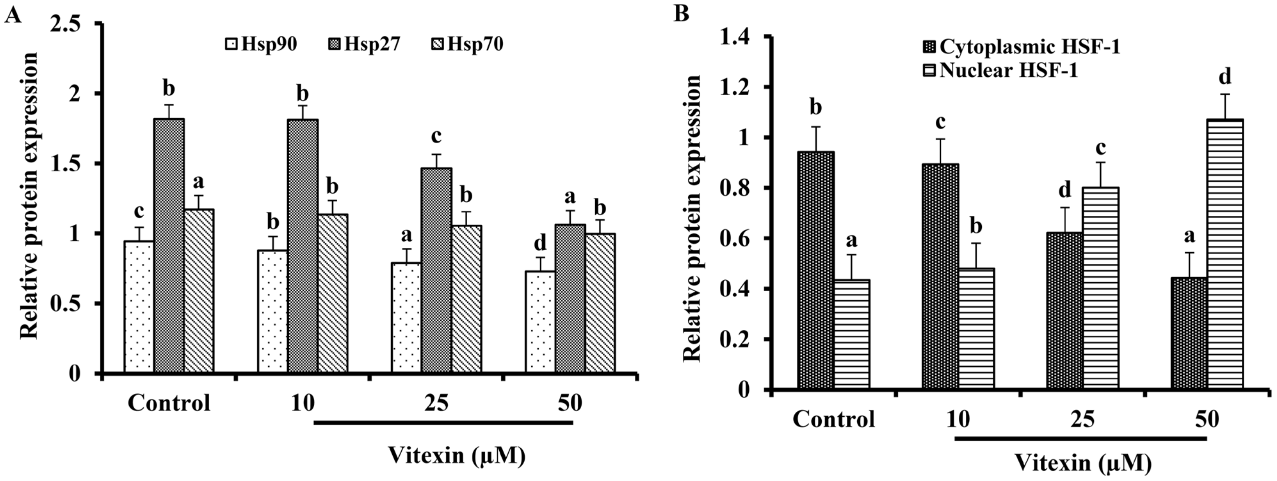

Supplementary Figure 3: The effects of vitexin on HCT-116 cell survival. (**A**) Representative quantitative immunoblot analysis of Hsp90, Hsp27 and Hsp70 proteins were determined by ImageJ. β-actin was used as loading control. (**B**) Representative quantitative immunoblot analysis of HSF-1 from Cytosolic and nuclear fractions were determined by ImageJ. β-actin and Lamin B were used as loading control for cytoplasm and nuclear blots respectively. The data are represented as mean ± SD of three independent experiments, $n = 3$. Values with different letters (a-d) differ significantly from each other ($p < 0.05$).

A

	Control	Vitexin (μM) 10	25	50
Intensity	53817	45556	36408	26422

B

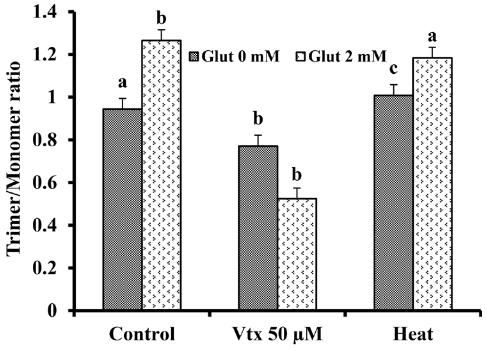

C

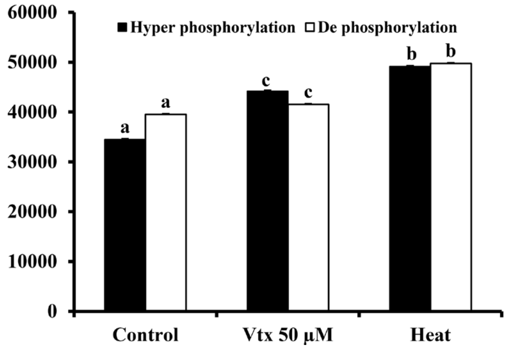

Supplementary Figure 4: (**A**) EMSA gel shift band intensity as quantified by software ImageJ. Quantification of HSF-1 (**B**) oligomerization and (**C**) phosphorylation were determined by ImageJ. β-actin was used as loading control for phosphorylation assay. The data are represented as mean ± SD of three independent experiments, $n = 3$. Values with different letters (a-c) differ significantly from each other ($p < 0.05$).

A

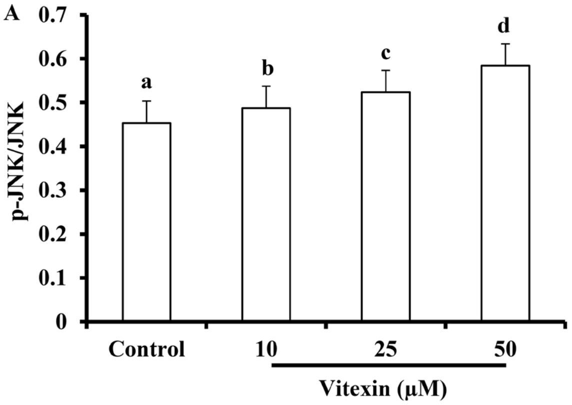

B

	Control	Vitexin (μM) 10	25	50
Intensity	22541	28735	32042	35980

C

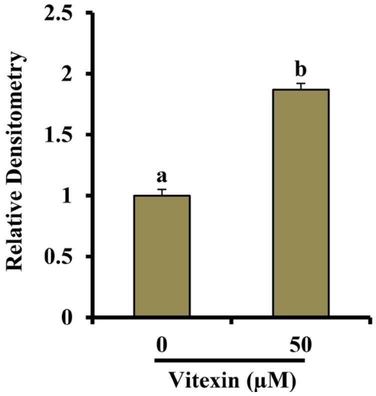

Supplementary Figure 5: (**A**) Ratio of p-JNK/JNK was quantified by ImageJ. β-actin was used as loading control. (**B**) EMSA gel shift band intensity as quantified by software ImageJ. (**C**) Relative densitometry of JNK in HSF-1 immunoprecipitates were determined by ImageJ. HSF-1 was used as loading control. The data are represented as mean ± SD of three independent experiments, $n = 3$. Values with different letters (a-d) differ significantly from each other ($p < 0.05$).

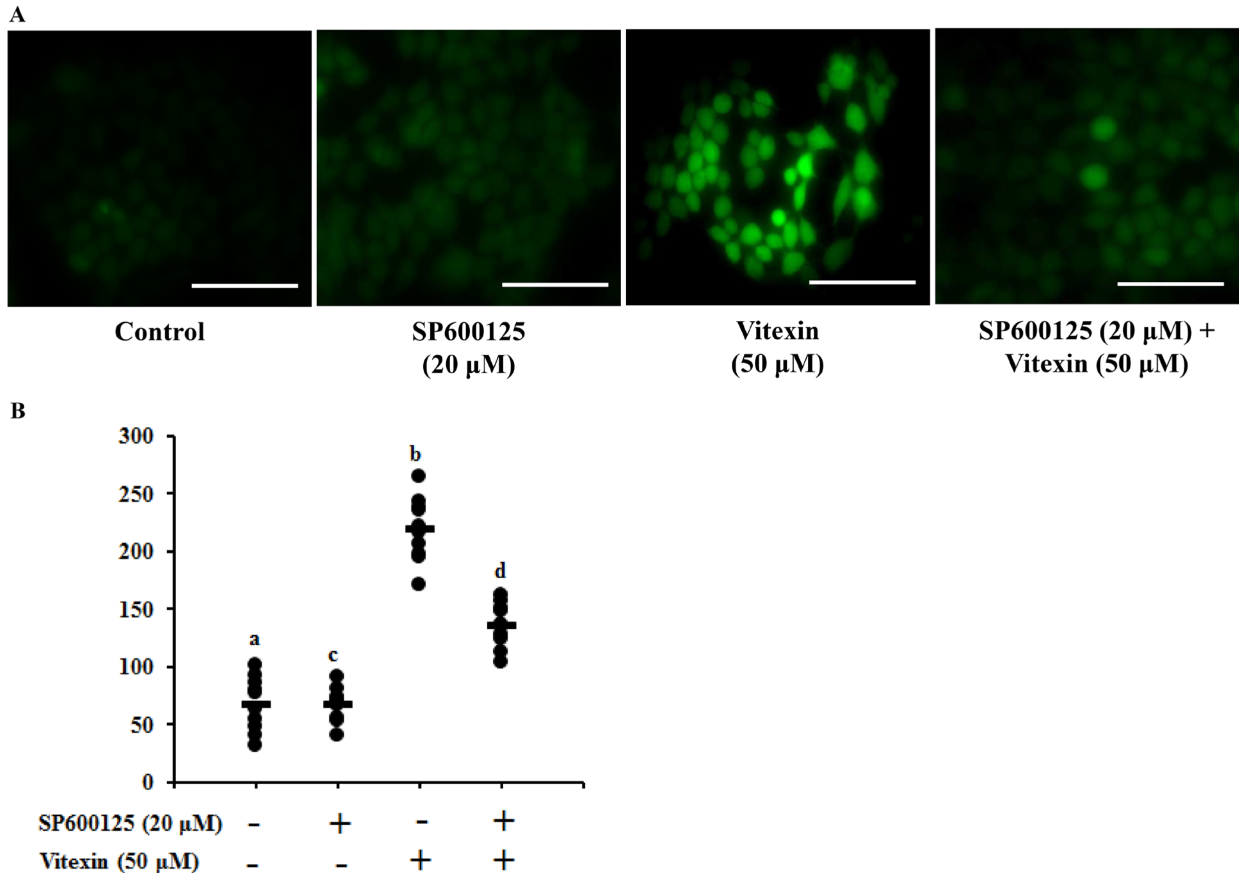

Supplementary Figure 6: (**A**) Cells were loaded with H2DCFDA for 30 mins and ROS levels were determined by fluorescence microscopy in different groups treated alone or together in the presence of JNK inhibitor SP600125. Scale bar = 0.1 mm. (**B**) Representative images and quantitative analysis of ROS generation were determined by ImageJ. The data are represented as mean ± SD of three independent experiments, *n* = 3. Values with different letters (a-d) differ significantly from each other ($p < 0.05$).

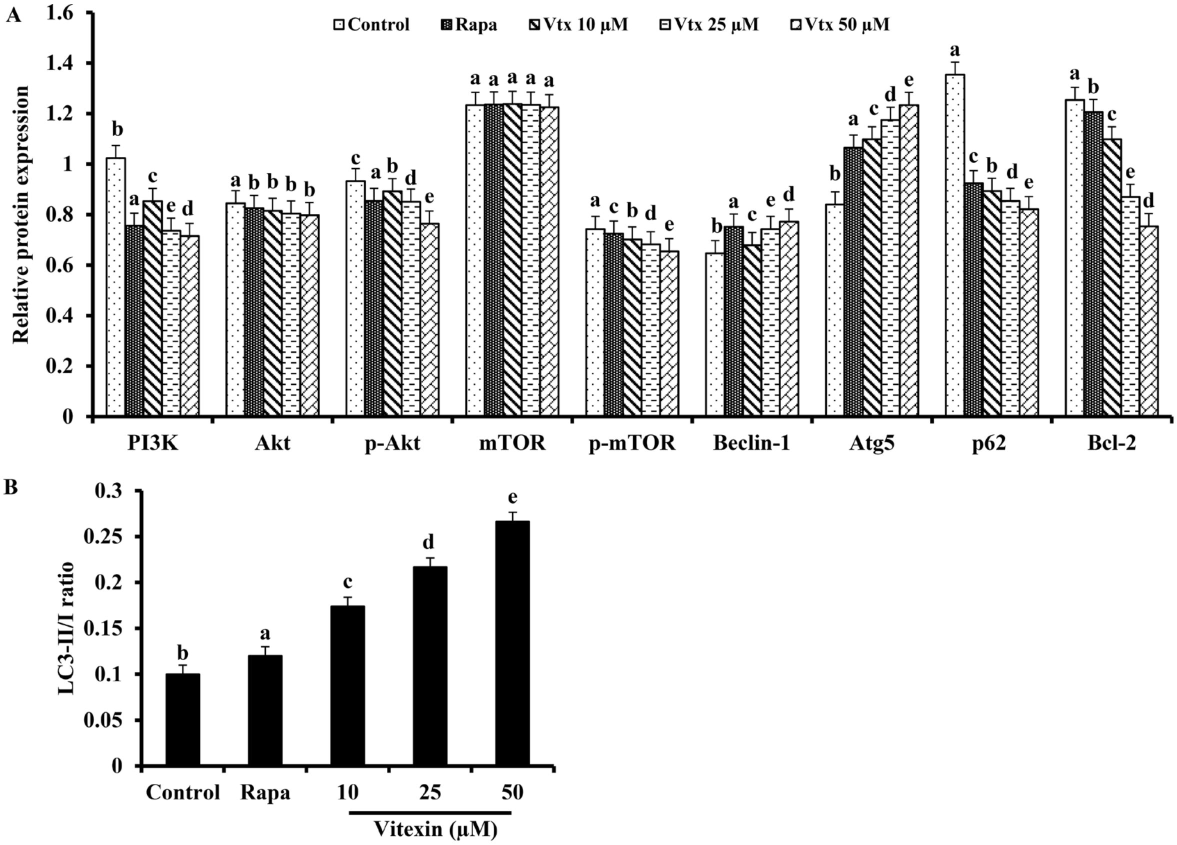

Supplementary Figure 7: (**A**) Quantification of immunoblot analysis for autophagy marker proteins was performed using ImageJ. β-actin was used as loading control. (**B**) The LC3-II/-I ratio was calculated on the basis of densitometric measurements of the Western blot signals. The data are represented as mean ± SD of three independent experiments, n = 3. Values with different letters (a-e) differ significantly from each other ($p < 0.05$).

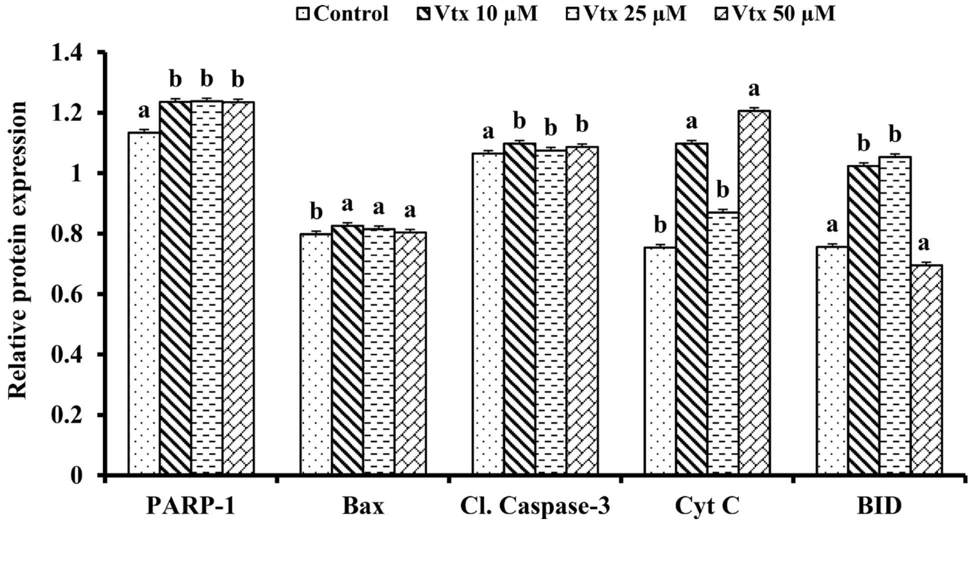

Supplementary Figure 8: Quantification of immunoblot analysis for apoptotic marker proteins was performed using ImageJ. β-actin was used as loading control. The data are represented as mean ± SD of three independent experiments, n = 3. Values with different letters (a-b) differ significantly from each other ($p < 0.05$).

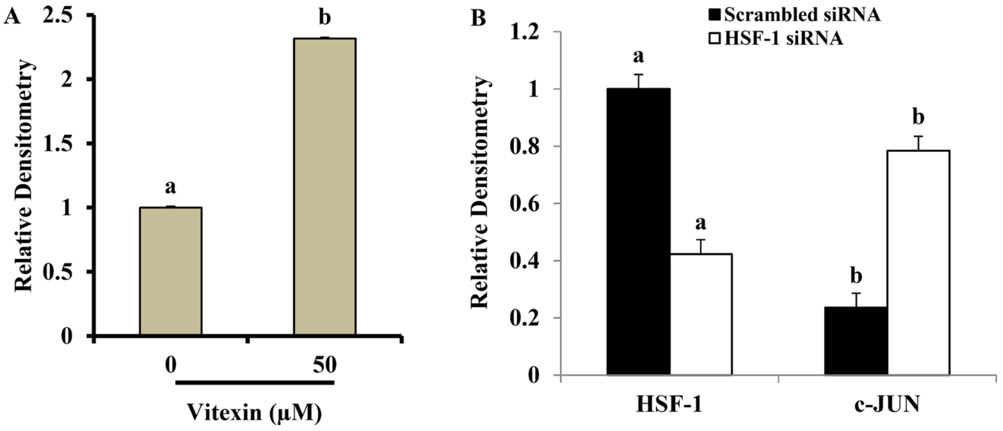

Supplementary Figure 9: (**A**) Relative densitometry of ApoL1 in HSF-1 immunoprecipitates were determined by ImageJ. HSF-1 was used as loading control. (**B**) Relative densitometry of HSF-1 and c-JUN in scrambled and HSF-1 silenced cells was performed by ImageJ. β-actin was used as loading control. The data are represented as mean ± SD of three independent experiments, $n = 3$. Values with different letters (a-b) differ significantly from each other ($p < 0.05$).

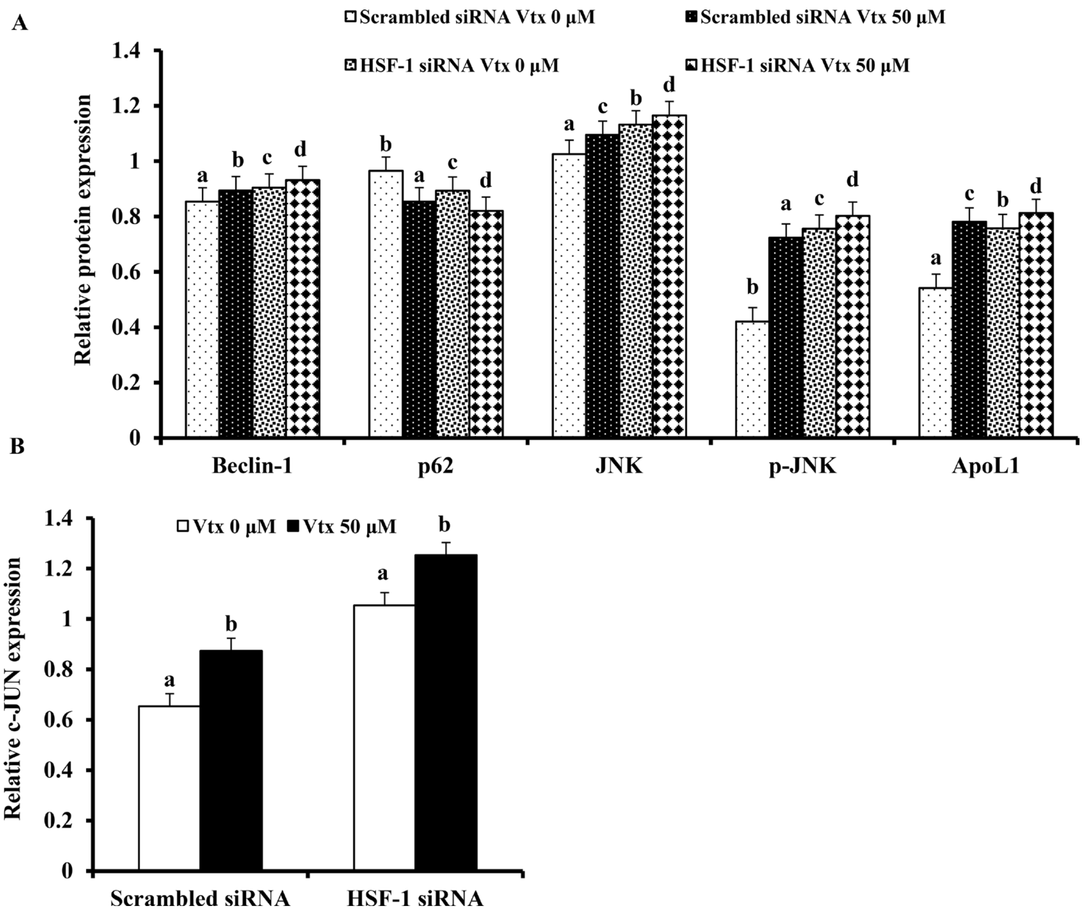

Supplementary Figure 10: (**A**) Quantification of immunoblot analysis for Beclin-1, p62, JNK, p-JNK and ApoL1 proteins were performed using ImageJ. β-actin was used as loading control. (**B**) Relative c-JUN expression after vitexin treatment in scrambled and HSF-1 silenced cells was performed by ImageJ. Lamin B was used as loading control. The data are represented as mean ± SD of three independent experiments, $n = 3$. Values with different letters (a-d) differ significantly from each other ($p < 0.05$).

Supplementary Table 1: List of primary antibodies

Antigen	WB dilutions	IP dilutions	Antibody name	Catalog number	Company
Actin	1:1000	——	Actin (I-19) Goat pAb	sc-1616	SCB
Hsp90	1:1000	——	HSP 90 Mouse mAb	ADI-SPA-830	ELS
Hsp27	1:1000	——	HSP 27 (C-20) Goat pAb	sc-1048	SCB
Hsp 70	1:1000	——	HSP 70 (K-20) Goat pAb	sc-1060	SCB
ApoL1	1:1000	——	apoL1 (A-3) Mouse mAb	sc-390440	SCB
Lamin B	1:1000	——	Lamin B (M-20) Goat pAb	sc-6217	SCB
PARP-1	1:1000	——	PARP-1 (H-250) Rabbit pAb	Sc-7150	SCB
LC3	1:1000	——	Anti-LC3A/B Rabbit pAb	Ab58610	Abcam
Beclin-1	1:1000	——	Beclin-1 (D40C5) Rabbit mAb	#3495	CST
p62	1:1000	——	SQSTM1/p62 Rabbit pAb	#5114	CST
JNK	1:1000	——	JNK (FL) Rabbit pAb	sc-571	SCB
p-JNK	1:1000	——	p-JNK (Thr 183/Tyr 185) Goat pAb	sc-12882	SCB
c-Jun	1:1000	——	c-Jun (D) Rabbit pAb	sc-44	SCB
Bcl2	1:1000	——	Bcl2 (N-19) Rabbit pAb	sc-492	SCB
PI3K	1:1000	——	Anti-PI3 Kinase, p85, N-SH2, clone UB93-3	05-217	Millipore
Atg5	1:1000	——	Atg5 (D5F5U) Rabbit mAb	#12994	CST
BID	1:1000	——	BID (FL-195) Rabbit pAb	sc-11423	SCB
Caspase-9	1:1000	——	Caspase-9 p10 (H-83) Rabbit pAb	sc-7885	SCB
Caspase-3	1:1000	——	Caspase-3 (H-277) Rabbit pAb	sc-7148	SCB
Bax	1:1000	——	Bax (N-20) Rabbit pAb	sc-493	SCB
Cyt-c	1:1000	——	Cytochrome c (H-104) Rabbit pAb	sc-7159	SCB

WB, western blot; IP, immunoprecipitation; SCB, Santa Cruz Biotechnology; ELS, Enzo Life Sciences; CST, Cell Signaling Technology

Antigen	WB dilutions	IP dilutions	Antibody name	Catalog number	Company
mTOR	1:1000	——	mTOR (H-266) Rabbit pAb	sc-8319	SCB
p-mTOR	1:1000	——	p-mTOR (Ser 2448) Rabbit pAb	sc-101738	SCB
Akt 1/2	1:1000	——	Akt1/2/3 (H-136) Rabbit pAb	sc-8312	SCB
p-Akt1/2	1:1000	——	p-Akt1/2/3 (Ser 473)-R Rabbit pAb	sc-7985-R	SCB
HSF-1	1:1000	5µg/500 µg total protein<br>**IF dilutions** 1:200	HSF-1 (H-311) Rabbit pAb	sc-9144	SCB

WB, western blot; IP, immunoprecipitation; IF, immunofluorescence; SCB, Santa Cruz Biotechnology; ELS, Enzo Life Sciences; CST, Cell Signaling Technology

Supplementary Table 2: List of secondary antibodies

Antigen	WB dilutions	Antibody name	Catalog number	Company
Rabbit IgG secondary antibody	1:10000	Goat anti-rabbit IgG-HRP	sc-2004	SCB
Goat IgG secondary antibody	1:5000	Donkey anti-goat IgG-HRP	sc-2020	SCB
Mouse IgG secondary antibody	1:5000	Goat Anti-mouse IgG-HRP	sc-2005	SCB
Goat Anti-Rabbit IgG	**IF dilutions** 1:400	Goat Anti-Rabbit IgG-FITC	sc-2012	SCB

WB, western blot; IF, immunofluorescence; SCB, Santa Cruz Biotechnology

Supplementary Table 3: List of oligonucleotide pairs for EMSA

Gene	Sequence (5′ to 3′)	size
**HSE**	CTAGAAGCTTCTAGAAGCTTCTAG	24
**AP1**	Forward: CGCTTGATGACTCAGCCGGAA	21
	Reward: TTCCGGCTGAGTCATCAAGCG	

Supplementary Table 4: List of primer pairs for qRT-PCR

Gene	Sequence (5′ to 3′)	size
β-actin	Forward: AATCTTCCGCCTTAATACTTC	21
	Reward: TATTGGTCTCAAGTCAGTGTA	
hprt	Forward: GTTGGGCTTACCTCACTGCT	20
	Reward: TAATCACGACGCTGGGACTG	
hsp90	Forward: TGAAGATGTTGGTTCTGATG	20
	Reward: TTCTCCGTACTCCTCATTAG	
hsp27	Forward: CGCCAAGTAAAGCCTTAG	18
	Reward: GGTGGTTGCTTTGAACTT	
hsp70	Forward: TGTCGTCCAGCACCCAGGCCAGC	22
	Reward: GCTCTTGTTCAGGTCGCGCCC	

Supplementary Table 5: List of siRNA sequence

SiRNA	Sequence (5′ to 3′)
**HSF-1**	Sense: CAGGUUGUUCAUAGUCAGAAUUGTA
	Antisense: ACGUCCAACAAGUAUCAGUCUUAACAU
